# Study protocol for a randomized controlled trial of in-home decluttering augmentation of group cognitive-behavioral therapy for hoarding disorder: the Joining Forces Trial

**DOI:** 10.1186/s13063-023-07509-4

**Published:** 2023-07-29

**Authors:** Sofia Jägholm, Sara Lindstedt, Erik Andersson, David Mataix-Cols, Lorena Fernández de la Cruz, Christian Rück, Volen Z. Ivanov

**Affiliations:** 1 Centre for Psychiatry Research, Department of Clinical Neuroscience, Karolinska Institutet, M48, Karolinska Universitetssjukhuset Huddinge, 14186 Stockholm, Sweden; 2 Stockholm Health Care Services, Region Stockholm, Stockholm, Sweden; 3 Division of Psychology, Karolinska Institutet, Nobelsväg 9, 17195 Solna, Sweden; 4 Centre for Psychiatry Research, Department of Clinical Neuroscience, Karolinska Institutet, Gävlegatan 22, 113 30 Stockholm, Sweden

**Keywords:** Compulsive hoarding, Hoarding disorder, Cognitive behavioral therapy, Home visits, De-cluttering, Study protocol, Randomized controlled trial

## Abstract

**Background:**

Cognitive behavioral therapy (CBT) is a moderately efficacious treatment for hoarding disorder (HD), with most individuals remaining symptomatic after treatment. The Joining Forces Trial will evaluate whether 10 weeks of in-home decluttering can significantly augment the outcomes of group CBT.

**Methods:**

A randomized controlled trial of in-home decluttering augmentation of group CBT for HD. Adult participants with HD (*N* = 90) will receive 12 weeks of protocol-based group CBT for HD. After group CBT, participants will be randomized to either 10 weeks of in-home decluttering led by a social services team or a waitlist. The primary endpoint is 10 weeks post-randomization. The primary outcome measures are the self-reported Saving Inventory-Revised and the blind assessor-rated Clutter Image Rating. Participants on the waitlist will cross over to receive the in-home decluttering intervention after the primary endpoint. Data will be analyzed according to intention-to-treat principles. We will also evaluate the cost-effectiveness of this intervention from both healthcare and societal perspectives.

**Discussion:**

HD is challenging to treat with conventional psychological treatments. We hypothesize that in-home decluttering sessions carried out by personnel in social services will be an efficacious and cost-effective augmentation strategy of group CBT for HD. Recruitment started in January 2021, and the final participant is expected to reach the primary endpoint in December 2024.

**Trail registration:**

ClinicalTrials.gov NCT04712474. Registered on 15 January 2021

**Supplementary Information:**

The online version contains supplementary material available at 10.1186/s13063-023-07509-4.

## Background

Hoarding disorder (HD) is a mental disorder that is present in approximately 2.5% of the general population [1]. It is characterized by significant difficulties in discarding or parting with possessions and strong urges to save things [2]. Individuals with HD often also have a strong desire to acquire items. Together, these symptoms result in the accumulation of large amounts of items that are kept in a disorganized manner at home and clutter the space to a degree that normal use of the living space is difficult, causing significant distress and impairment in function [2]. HD is considered difficult to treat and often requires a coordinated response from the community [3].

The diagnosis of HD was included as a separate mental disorder in 2013 in the latest edition of the Diagnostic and Statistical Manual of Mental Disorders (DSM–5) [2] and more recently in the International Classification of Diseases (ICD-11) [4]. Research into evidence-based treatments for HD is still in its infancy, but several interventions based on the principles of cognitive behavioral therapy (CBT) have been developed and evaluated during the last decade [5–8]. CBT for HD focuses on enhancing problem solving and organizational skills, handling emotions, exposure to discarding and not acquiring possessions, cognitive restructuring of hoarding-related beliefs, and increasing/maintaining motivation. A meta-analysis of HD trials showed large within-group effect sizes for CBT, regardless of modality (group vs. individual) [8]. However, for the vast majority of individuals with HD, impairing symptoms remain after treatment. This is particularly notable in the case of clutter, which has typically accumulated over several decades [8]. This indicates that new treatments or augmentation strategies are needed to further improve treatment outcomes and functioning.

A myriad of public and private de-cluttering services has proliferated in recent years, but their efficacy in reducing clutter and improving the outcomes of individuals with HD is unknown. One small pilot trial found that adding home visits, including sorting and discarding exercises, to CBT for HD decreased the overall clutter level in the homes of individuals with HD [9]. The intervention was described as feasible and well tolerated, and study participants also reported improvement in activities of daily living [9]. These encouraging results suggest that in-home decluttering may be a viable augmentation strategy to CBT for HD.

The Joining Forces Trial was designed by mental health professionals in partnership with social services to formally evaluate the additive effect of group CBT and in-home decluttering for adults with HD. Specifically, in this trial, we will evaluate whether in-home decluttering augmentation of group CBT is associated with decreased hoarding symptom severity, clutter, caregiver burden, and family accommodation of hoarding behaviors, as well as increased daily self-care activities. As a secondary aim, we will evaluate the cost-effectiveness of the intervention, which incorporates both a treatment provider perspective (i.e., direct costs such as costs for healthcare personnel) and a broader societal perspective (i.e., indirect costs such as sick leave). The current paper describes the rationale and methods of the study. The full study protocol can be found in the Supplement.

## Methods

### Study design and setting

**Fig. 1:**
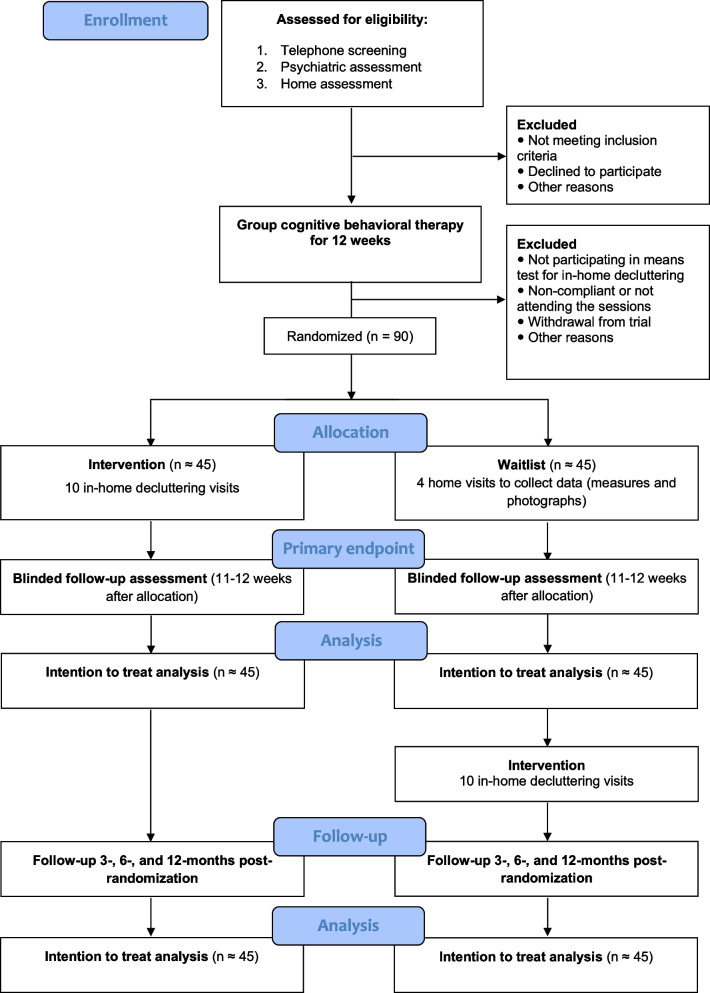
CONSORT diagram of the trial procedures

This study is a parallel-group, superiority trial. All study participants will first undergo protocol-based group CBT [10] for 12 weeks, and within 10 days after the end of the CBT intervention, they will be randomly assigned (1:1 ratio) to either 10 weeks of in-home decluttering or a waitlist. Participants randomized to the waitlist will be offered in-home decluttering at 10 weeks post-randomization (i.e., after the primary endpoint). All participants will be followed up naturalistically 3, 6, and 12 months after the primary endpoint. Figure 1 shows a CONSORT flow diagram [11] of the study design. A SPIRIT checklist [12] can be found in the supplements.

The study will be conducted in Stockholm, Sweden. The group CBT will be administered at *OCD-programmet*, a psychiatric outpatient unit within the publicly funded Stockholm County Council, specialized in obsessive-compulsive and related disorders. The in-home decluttering will be delivered by a specialized hoarding team within the social services in the municipality of Stockholm, Sweden.

### Participants

We will randomize 90 participants to either in-home decluttering or a waitlist. As we expect some participants to drop out from the group CBT prior to randomization, we will recruit participants to CBT until we have randomized 90 of them. We will advertise the trial to clinics and within the social services in Stockholm, patient organizations, and directly to the public via a designated trial website and social media. Participants can either self-refer or be referred to the trial via regular healthcare services.

Potential participants will initially be contacted via telephone by a member of the research team or, in some cases, approached face-to-face at the clinic. The main purpose of this screening is to assess potential eligibility, which includes asking brief questions about hoarding symptoms using the Hoarding Rating Scale – Interview (HRS-I) [13] and each inclusion/exclusion criterion. The inclusion and exclusion criteria are listed in Table 1. If potentially eligible and interested in taking part in the trial, the participants will be booked for a psychiatric assessment, which will be carried out by a psychologist or psychiatrist at the specialist clinic. The aim of this/these visit(s) is to (a) verify that diagnostic criteria for HD (except clutter) are met and (b) quantify the severity of the hoarding symptoms. The Structured Interview for Hoarding Disorder (SIHD) [14] will be administered to assess diagnostic criteria for HD. The Mini International Neuropsychiatric Interview [15] and a short diagnostic interview, the OCD-RD, will be administered to assess psychiatric comorbidities. The OCD-RD is originally based on the Structured Clinical Interview (SCID) for DSM-5 and assesses all the disorders in the OCD and related disorders chapter [16].

**Table 1:** Eligibility criteria

**Inclusion criteria**	**Exclusion criteria**
1. Age 18 years or older 2. HD as a primary psychiatric diagnosis 3. Willing and able to understand and provide consent and complete the study procedures 4. Citizen in the municipality of Stockholm or living within a 1-h commute by public transport from the social services office delivering the in-home decluttering intervention and eligible for social assistance according to the Stockholm social services	1. Concurrent CBT or having received CBT for HD during the last two years for a minimum of 8 sessions, including active strategies for reducing acquisition and practice of discarding with a qualified therapist *or* 8 previous in-home decluttering sessions with a qualified social worker 2. Unable or unwilling to allow the study staff into their home for home assessment 3. Animal hoarding or squalid (i.e., extremely unhygienic) home conditions that are deemed to put the personnel at risk during the in-home decluttering 4. A diagnosis of organic brain disorder, intellectual disability, psychosis, anorexia nervosa, alcohol/substance dependence or abuse, or bipolar disorder without stable medication or with symptoms during the last 6 months 5. Major medical or neurological conditions that would prevent completing assigned behavioral practice tasks 6. Immediate risk to self or others requiring urgent medical attention, such as high suicidality risk 7. Not able to read and communicate in Swedish 8. Currently at high risk of eviction (for instance having received an eviction notice from a housing company or Swedish court) 9. Living in the same household as an already included participant

Some of the exclusion criteria, such as the presence of squalor in the home, will be assessed during a home assessment, which will follow the psychiatric assessment. The home assessment is carried out by one psychologist and one social worker and aims to confirm the HD diagnosis with regard to the amount of clutter and to determine whether delivering in-home decluttering would be feasible.

If all inclusion criteria and no exclusion criteria are met, the participant signs the informed consent during the home assessment and is preliminarily included in the trial. The consent is obtained by the psychologist on delegation of the principal investigator. The final step of the inclusion procedure consists of a “means test” from the social services unit regarding the in-home decluttering. The means test includes an evaluation of the participant’s individual needs for social assistance, for instance, outreach housing support, according to the Swedish Social Services Act [17]. This act aims to ensure the right to a reasonable standard of living for people who live in Sweden. A person with a physical or psychiatric disability who cannot meet this standard on their own and has had a disability for more than 2 years is eligible for such assistance.

#### Relatives and relevant persons

The level of care burden experienced by relatives of people with HD is high [18], but no support is typically available for this population. Although the interventions in the current trial are targeted at individuals with HD, we want to explore whether they might have a wider beneficial impact on their relatives. Therefore, before enrolling in the trial, all participants will be asked if they have a relative (or other significant persons) that may be contacted by the researchers. If the participant consents to this, this relative will receive information about the trial and questionnaires by mail and be asked to complete the questionnaires before the start of group CBT, before the start of the in-home decluttering (or waitlist), and at the primary endpoint. The relative will be informed that study participation is voluntary and sign a consent form.

#### Concurrent interventions

Participants are free to continue any medication during the trial. As there is no well-established pharmacological treatment for HD [19, 20], the participants are allowed to make changes to their psychiatric medication regime for the duration of the trial. Potential medication changes will be noted in the follow-up assessments. Concurrent interventions from the social services, such as outreach housing support, will be allowed if this intervention does not focus on the participants’ hoarding difficulties or clutter. This will be confirmed and coordinated by the study personnel after inclusion, either through a face-to-face or a phone meeting with the corresponding social services. Furthermore, the participants are asked to not start any parallel/new psychological treatment (of any type) for the indication of HD until after the 3-month follow-up.

### Interventions

#### Group cognitive-behavioral therapy for hoarding disorder

All included study participants will first receive group CBT for HD, delivered at the specialist outpatient unit. We aim to include 8 patients in each group, but the number of group members can vary from 5 to 9 depending on the recruitment rate and staffing and scheduling possibilities of the outpatient unit. Group CBT will be based on a published manual [10]. The original manual includes 16 1.5-h weekly sessions. However, in the current trial, the treatment will be delivered in 12 2-h weekly sessions. All components from the original manual will thus be delivered in full (24 therapy hours), albeit during a shorter time frame. The shortening of the time frame is to mitigate the risk of treatment fatigue among the participants due to the long nature of the combined intervention (i.e., CBT + in-home decluttering). Evidence from one study suggests that the efficacy of this shorter form of CBT (i.e., 12 sessions) is similar to that of a longer-duration group and individual CBT for HD [21]. Each session will be facilitated by two psychologists, of which at least one has previous experience in treating HD.

The CBT manual includes psychoeducation about CBT and HD, goal setting, executive skills training, exposure to discarding and response prevention (practice not acquiring possessions in high-risk situations that trigger an urge to acquire), cognitive restructuring, mindfulness-based skills to accept and tolerate negative emotions, motivation enhancement, learning how to combine these components, and relapse prevention. Group sessions start with a review of the homework assignment from the previous week, followed by a review and exercises regarding a specific hoarding-related topic. The efficacy of the treatment based on this manual was evaluated in a large randomized controlled trial [22], and our research group has tested the feasibility of delivering the treatment in the Swedish healthcare context [23]. Therapist adherence to the treatment protocol will be monitored using a checklist of administered treatment components after each session, filled out by one of the treating psychologists.

#### In-home decluttering

After the last group CBT session, an additional individual session will take place, during which the study participant will meet with one of the group facilitators (psychologists) and one of the social workers who will be providing the in-home decluttering service. During this meeting, the study participant’s CBT treatment and progress will be jointly reviewed and a detailed plan for the upcoming home visits be made. This session occurs directly after the CBT regardless of randomization outcome.

The in-home decluttering begins within 10 days after randomization and will be delivered by social workers. It includes 10 weekly 1.5-h home visits. These home visits are personalized, agenda-driven, goal-oriented, structured, and focused on decluttering. They include (1) a brief check-in, (2) guided unclutter time (including motivational enhancement, cognitive exercises, and exposure to aversive emotions), (3) a reflective period in which participants share their thoughts and objectives for the coming week, and (4) homework assignments. The content in the home visits thus mirrors the format that the study participants are already familiar with through the CBT. However, the purpose of the home visits is to focus on practical management of clutter (i.e., removing and reorganizing) with an emphasis on improving the participants’ ability to perform daily activities in their home (e.g., clearing the kitchen to be able to cook and the sofa to be able to invite visitors).

Prior to the start of the decluttering intervention, all staff (social workers) who deliver in-home decluttering will read a treatment manual, specifically developed for this trial, and will undergo a 1-day workshop delivered by an expert in HD (VZI) and members from the social service team specialized in decluttering. The content of this workshop will focus on managing HD, strategies for delivering in-home decluttering, and study procedures. The members of the staff who have worked with individuals with hoarding for longer than 6 months will not be offered the workshop but will receive a thorough review of the study procedures. During the trial, the staff will receive regular supervision by experts on HD. Adherence to the in-home decluttering protocol will be monitored using a checklist of administered components after each session, filled out by one of the social workers. The criteria for noncompliance and handling procedures during both the group CBT and the in-home decluttering are described in the supplemented full protocol.

#### Waitlist

Participants assigned to the waitlist will receive no in-home decluttering for a period of 10 weeks after group CBT. During this period, a study assessor will visit the participants every third week to ask them to complete the pertinent assessment measures and have photographs of the home taken for blinded CIR ratings. Audio from all home visits will be recorded and monitored by supervisors to ensure that no unintended interventions were given during these visits. After these 10 weeks (primary endpoint), participants on the waitlist will be crossed over and offered the same intervention as the active group.

### Randomization

After group CBT, study participants will be randomized (1:1 ratio) to either 10 weeks of in-home decluttering or a waitlist of identical duration. The randomization sequence will be generated before the randomization of the first participant using masked block randomization by an independent clinical trials unit not involved in the study (Karolinska Trial Alliance; https://karolinskatrialalliance.se). Randomly varying block sizes will be generated using a computer random number generator. Participants will be informed of group assignments at the meeting with the social services and the CBT facilitator, which takes place after group CBT and before in-home decluttering.

### Outcome measures

**Fig. 2:**
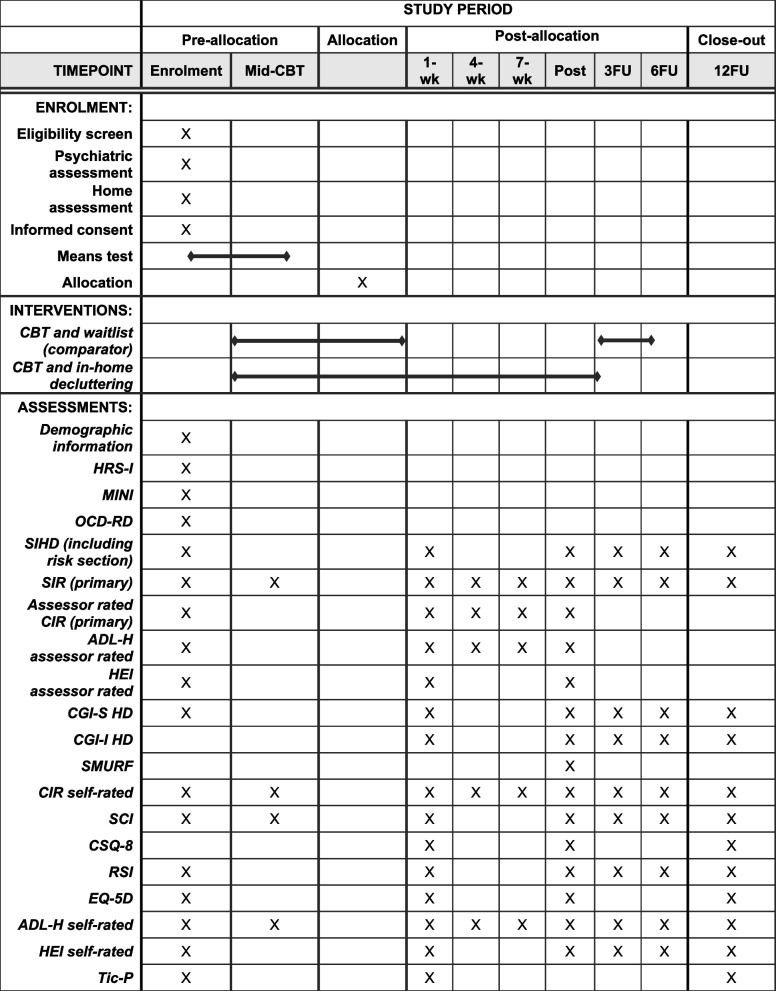
SPIRIT schedule of enrollment, interventions, and assessments. Abbreviations: CBT, cognitive behavioral therapy; 1-wk, before or during the first home visit, the equivalent of the baseline of in-home decluttering or waitlist; 4-wk-7wk, assessment points 4 and 7 weeks post-randomization; post, 10 weeks after starting the intervention, the primary endpoint; 3FU-12FU, assessment points 3–12 months after the primary endpoint; HRS-I, Hoarding Rating Scale – Interview; MINI, Mini International Neuropsychiatric Interview; OCD-RD, part from the structured Clinical Interview that assesses all the disorders in the OCD and related disorders; SIHD, Structured Interview for Hoarding Disorder; SI-R, Saving Inventory-Revised; CIR, Clutter Image Rating; ADL-H, Activities of Daily Living for Hoarding; HEI, Home Environment Index; CGI-S, Clinical Global Impression Scale Severity; CGI-I, Clinical Global Impression Scale Improvement; SMURF, Safety Monitoring Uniform Report Form; SCI, Saving Cognitions Inventory; CSQ-8, Client Satisfaction Questionnaire-8; RSI, Relationship between Self and Items; EQ-5D, EuroQol-5D; TiC-P, Trimbos Questionnaire for Costs associated with Psychiatric Illness

Assessments will be conducted at several time points throughout the trial. All measures and measurement points are shown in Fig. 2; for more details, see supplement 1 (full protocol). Baseline assessment occurs after CBT, and during the first home visit of in-home decluttering or waitlist. Two mid-intervention assessments will be done, one at week 4 and the other at week 7 after randomization. The primary endpoint is 10 weeks after randomization.

#### Primary outcome measures

The primary outcome measures are the self-reported Saving Inventory-Revised (SI-R) [24] and the blind assessor-rated Clutter Image Rating (CIR) [25]. The SI-R is a 23-item questionnaire assessing the severity of hoarding symptoms, including the three subscales difficulty discarding, clutter, and excessive acquisition. Each item is rated from 0 to 4, with a higher number indicating more symptoms of HD; all items are summed to generate a total score. The SI-R is one of the most widely used self-rated measures of HD and has strong psychometric properties and a high discriminant performance [26].

The CIR is a visual scale assessing the levels of clutter in different rooms in the home, using photographs with 9 levels of clutter, and has sound psychometric characteristics [25, 27]. During the in-home decluttering, the social workers will collect the administered measurements. For waitlisted participants, a study assessor will visit the participants’ homes every third week (corresponding to intervention weeks 1, 4, 7, and 10 in the intervention group) in order to complete the pertinent assessment measures. The assessors and the social workers will take photographs of the participants’ homes, which will be rated with the CIR by blind assessors not involved in any parts of the interventions in the trial.

#### Secondary outcome measures

Secondary blinded assessor-rated outcome measures include the Clinical Global Impression Scale (CGI) [28], a brief assessment of the clinician’s view of the patient’s HD severity and improvement. It consists of measures of the severity of illness (CGI-S) and improvement after treatment (CGI-I). Both measures will assess severity and impairment in relation to the HD symptoms only.

Participants will fill out the self-reported version of the CIR [25]. They will also self-rate their cognitions about discarding their possessions using the Saving Cognitions Inventory (SCI) [29] and their level of object attachment by using the visual Relationship between Self and Items (RSI) [30], which is an adaptation of the Inclusion of Other in Self scale [31]. The self-administered EuroQol-5D [32] provides a single index value that will be used in both clinical and economic evaluations of treatment and health care. The Client Satisfaction Questionnaire-8 (CSQ-8) [33] will be used to assess clients’ satisfaction with the in-home decluttering. The Trimbos Questionnaire for Costs associated with Psychiatric Illness (TiC- P) [34] will be used to assess healthcare and societal resource use.

The following secondary measures will be administered as both clinician- and participant-rated outcomes: the Activities of Daily Living for Hoarding (ADL-H) [35], measuring the degree to which hoarding symptoms impair daily functioning, and the Home Environment Index (HEI) [36], measuring the sanitary state of the participant’s homes. Since follow-up assessments will be carried out at the psychiatric clinic, and not in the participant’s homes, the ADL-H and the HEI will solely be administered as self-report forms during these assessment points.

In line with previous studies of HD [37, 38], responder status will be operationalized as clinically significant change on SI-R (change of 14 points or more), and remitter status will be operationalized as being a responder and having a total score of SI-R below a cutoff of 42 [39].

The available relatives/significant persons of the participants will answer questions about demographic variables and will fill out the Family Impact Scale for Hoarding Disorder (FISH) [40], a scale that measures the level of family accommodation to the hoarding behaviors displayed by the relative who hoards and the burden these put on families, and the Caregiver Burden Inventory (CBI), a non-hoarding specific measure [41].

#### Recording and reporting of adverse events

Data on adverse events will be collected using a standardized checklist (Safety Monitoring Uniform Report Form, SMURF [42] at the primary endpoint. If the research team is notified about adverse events at other times, this will be logged, and if needed, an assessment about continued participation in the study will be done. Reported adverse events will be categorized depending on severity and frequency in line with Good Clinical Practice principles. Serious adverse events (i.e., events that result in death, suicide attempt, serious violent incident, or admission to hospital) will be documented and monitored by Karolinska Trial Alliance. If a participant expresses suicidal ideation or a worsening of symptoms is suspected, a clinical assessment including a structured suicide risk evaluation will be conducted and suitable measures will be taken to manage the symptoms.

### Blinding

Assessors conducting primary endpoint assessments will be blind to group allocation. Participants will also be reminded by their assessors not to reveal their arm allocation. To measure blinding integrity, the primary endpoint assessments will be audio recorded. All assessors will guess each participant’s intervention allocation at each assessment point and motivate their choice. They will also register whether the participants inadvertently revealed their group allocation. If that is the case, the audio recording will be edited, and a new blind assessor will listen to the recording and conduct the rating that will be used in the trial. The follow-up assessments will not be blinded, because all participants will have received, or at least been offered, the in-home decluttering by the first follow-up assessment (3-month follow-up). The statistician performing data analyses will be kept blind to participant group allocation during the whole duration of the trial.

### Patient and public involvement

This trial builds upon previous feedback from therapists, social workers, patients, and patient organizations with a desire to improve collaboration between healthcare and municipalities.

The Task Force for Hoarding Disorder in Stockholm has been and will continue to be a collaborating partner in this study. This ensures that experience from every phase of the project is directly communicated to all relevant interested parties (including patient representatives). Moreover, patients will be represented by the Swedish OCD patient organization (https://ocdforbundet.se/), which is a member of the task force, making sure that the service user perspective is acknowledged throughout all phases of the project.

Individuals that have received treatment for HD outside the context of the current study have continuously had the opportunity to provide feedback about their experiences. As a result of this feedback, some small modifications to the CBT workbook in Swedish were made (e.g., simplifying and making worksheets easier to use and using the word “treatment” instead of “course”). The patient organization for OCD and related disorders in Stockholm (*OCD-föreningen Stockholms län*; in English: OCD Association in Stockholm County) provided feedback on the current design and some modifications to the exclusion criteria were made accordingly.

### Data management

Data will be collected manually and digitally and kept in case report forms (CRFs). Each participant will receive a trial identification number, and their data will be handled according to rules of confidentiality. The accuracy of the data entry for the primary outcome measures (self-reported SI-R and blind assessor-rated CIR) will be monitored by the Karolinska Trial Alliance, and a delegation log for staff involved in data handling will be used.

### Sample size calculation and statistical analysis

The power calculation in the current trial is based on individual-level data from a previous study of group CBT for HD with the SI-R as the primary outcome measure, rated at 3 time points [23]. A linear, 2-level mixed effects model (LMM) implemented using the R package powerlmm, version 0.4 (available at https://cran.r-project.org/src/contrib/Archive/powerlmm/) [43] was used to calculate the required sample size. In this model, the standard deviations of the random intercept (4.22), the random slope (2.54), and the residual error (5.68) were imputed. Given 80% power (two-sided alpha test), two groups (in-home decluttering and waitlist), and 4 SI-R observations (baseline, week 4, week 7, post-intervention) per participant, we estimated that a total of 80 participants would be needed to detect a slope difference between the two groups of 8 points on the SI-R at the primary endpoint. For the other primary outcome measure, the blind assessor-rated CIR, data from the same study was used. In that study, there were only data from two measurement points, and therefore, an ANCOVA was used to calculate power, adjusting for pre-treatment scores. Given 80% power (two-sided alpha test), two groups (in-home decluttering and waitlist), and 4 CIR observations (baseline, week 4, week 7, post-intervention) per participant, the estimate is that a total of 58 participants would be needed to detect a slope difference between the two groups of 1 point on the CIR at the primary endpoint.

To compensate for up to 15% missing data throughout the trial, we will recruit a total of 90 participants who will be randomized to one of two trial arms. However, due to the risk of dropouts before randomization, during or immediately after CBT, we plan to recruit patients to CBT until we have randomized 90 participants.

All data will be analyzed according to intention-to-treat principles with mixed effects models. The mixed effect regression model will include fixed effects of time, trial arm (intervention vs. waitlist), and an interaction effect of time by trial arm, as well as a random intercept to account for individual differences. Ordinal variables (such as the CGI-I and the CGI-S) will be analyzed with ordinal regression and binary data (e.g., remitter status) with logistic regression. The chi-square tests will be used to check whether the blinded assessor’s guesses on trial arm allocation are better than chance. The selected statistical model of mixed effect regressions can handle missing data, if more than 15% of data is missing an appropriate imputation method will be applied. Potential selection bias due to missing data will be assessed by comparing data from enrollment and baseline from participants with and without missing data on the primary outcomes using linear regression and by conducting sensitivity analysis. The statistician that will perform the data analyses will be independent from the research group and blind to group allocation.

### Cost-effectiveness analysis

In the cost-effectiveness analysis, the costs and health outcomes of the participants in the in-home decluttering group will be calculated and compared to the same costs of the individuals randomized to the waitlist. We will first estimate the direct costs from a treatment provider perspective (e.g., resource use associated with home visits). We will then also include indirect costs, which will widen the scope to a societal perspective (e.g., sick leave or work absenteeism). We will perform both cost-effectiveness and cost-utility analyses. In the cost-effectiveness analyses, overall hoarding symptoms, responder and remitter status, and level of clutter, measured with the SI-R and the CIR, respectively, at the primary endpoint (10 weeks post-randomization) will be used as the health outcome. In the cost-utility analysis, the outcome will be the quality-adjusted life years (QALYs), according to international standards for cost-effectiveness analyses [44]. QALYs will be calculated using the area under the curve method. Costs will be estimated by appropriate regression analyses testing alternative link functions and distributions. Non-parametric bootstrapping with 1000 iterations will be carried out, pairing up differences in costs with differences in outcomes. As a global cost-effectiveness estimate, a visual presentation of cost-effectiveness planes will be presented. The timeframe for the cost-effectiveness analyses will be from baseline to the primary endpoint.

### Quality control

This study was preregistered (ClinicalTrials.gov reference number NCT04712474) prior to the inclusion of the first participant. External monitoring will ensure the good quality of the study procedures. The Karolinska Trial Alliance will monitor random samples of 15% of the study participants, focusing on informed consent forms, identification log, journal entry of consent forms, inclusion/exclusion criteria, primary outcome measures at the primary endpoint (i.e., SI-R and CIR), and serious adverse events. The study will also follow local regulations for data management and protection, the Swedish law, and the General Data Protection Regulation (GDPR).

### Ethics and dissemination

The Swedish Ethical Review Authority approved the study (reference numbers 2020-05798 and 2021-06176). From a clinical point of view, the proposed combined interventions pose a small risk to participants. All patients receive a thorough clinical assessment and those with more immediate needs or risks will be offered alternative treatment options. No patients will be denied current standard treatment (i.e., CBT for HD) due to drop-out from the study or if they do not want to enroll at all. Adverse events will be carefully monitored throughout the trial, but the risk of serious adverse events due to the interventions is considered very low. The adverse events that may be experienced in the short term, such as anxiety due to discarding belongings, are difficult to avoid and are often necessary for the participants to reduce their hoarding difficulties in the long run.

We tentatively plan to publish a main paper with the efficacy results and secondary papers with cost-effectiveness results and the naturalistic follow-up data. We also plan to disseminate the results of the research to study participants, the Swedish OCD Foundation (*Svenska OCD-förbundet*), members of the task force, politicians, and the general public. Results will also be presented at scientific conferences.

## Discussion

This trial will assess the efficacy and cost-effectiveness of in-home decluttering augmentation of group CBT for people with HD. A total of 90 adult participants with primary HD treated with group CBT will be randomized to either 10 home visits with an in-home decluttering component or to a waitlist. Data will be collected at multiple assessment points and with similar procedures for both groups. Outcome assessors will be blind until the primary endpoint. The waitlisted participants will be offered the in-home decluttering after the primary endpoint. All participants will be followed up to 12 months after the primary endpoint.

Practical issues that have occurred during the trial include disruptions caused by the COVID-19 pandemic. This trial has followed, and will continue to follow, local regulations and adapt/modify procedures according to these, if relevant. Other issues involve collaboration between several authorities (i.e., healthcare and social services) with different regulations and infrastructures. In order to solve such issues, changes to the first protocol version were made and documented, and amendments submitted for approval to the ethics committee.When interpreting the results, the potential limitations of the study design will be considered. The choice to use a waitlist as the control condition has limitations and may bias our estimate of the efficacy of in-home decluttering, as it is not compared to an alternative active intervention. However, during the waitlist period, participants will receive 4 short home visits with the purpose of data collection. Although no treatment will be delivered during these visits, the mere presence of an assessor in their homes may provide the participants with some sense of support and/or accountability for continuing to use CBT skills on their own. Finally, the cross-over design, in which the control group is offered in-home decluttering after the primary endpoint, will limit the conclusions that can be drawn for the long-term efficacy of the treatment, given that this follow-up will be naturalistic (i.e., uncontrolled). Nonetheless, we chose this design based on the ethical implications for the participants randomized to the waitlist.

## Conclusion

The unique features of HD pose challenges that often require a coordinated response from different authorities. Existing behavioral interventions for the disorder are somewhat efficacious but leave ample room for improvement. If our hypotheses are confirmed, the Joining Forces Trial has the potential to advance the field by providing a concrete model of collaboration between healthcare and social services for the management of HD.

## Trial status

Recruitment to the study started in January 2021 and is currently ongoing. At the time of submission of this paper (May 2023), a total of 48 participants had been randomized. According to the planned timeframe, recruitment will be ongoing until August 31, 2024, and all data from the primary endpoint will be collected by the end of 2024. The follow-up assessments will continue up to 1 year after the last participant has reached the primary endpoint. This paper was based on protocol version 4.1, see supplement.

## Supplementary Information

**Additional file 1.** Full study protocol version 4.1. 

**Additional file 2.** SPIRIT Checklist for Trials.

## Data Availability

The results are planned to be published in peer-reviewed scientific journals and presented to the media as well as at national and international conferences. Trial data could potentially be shared with other HD researchers around the world in accordance with laws and local regulations and whenever data sharing is permitted. The use will primarily be for similar objectives as in the current trial, for instance, to combine treatment outcome data for meta-analyses. Shared data will be pseudonymized. The possibility of future data sharing is mentioned in the participants’ informed consent form.

## Abbreviations

CBT: Cognitive behavioral therapy
HD: Hoarding disorder
HRS-I: Hoarding Rating Scale Interview
SIHD: Structured Interview for Hoarding Disorder
SCID: Structured Clinical Interview
SIR: Saving Inventory-Revised
CIR: Clutter Image Rating
CGI: Clinical Global Impression Scale
GCI-S: Clinical Global Impression Scale Severity
CGI-I: Clinical Global Impression Scale Improvement
SCI: Saving Cognitions Inventory
RSI: Self and Items
CSQ-8: Client Satisfaction Questionnaire-8
TiC-P: Trimbos Questionnaire for Costs associated with Psychiatric Illness
ADL-H: Activities of Daily Living for Hoarding
HEI: Home Environment Index
FISH: Family Impact Scale for Hoarding Disorder
CBI: Caregiver Burden Inventory
SMURF: Safety Monitoring Uniform Report Form
CRFs: Case report forms
LMM: Linear mixed effects model
QALY: Quality-adjusted life year
GDPR: General Data Protection Regulation
