# Supplementary material for: Study protocol for a randomized controlled trial of in-home decluttering augmentation of group cognitive-behavioral therapy for hoarding disorder: the Joining Forces Trial
Source: Trials. 2023 Jul 29;24:483. doi: 10.1186/s13063-023-07509-4 (PMC10386309; doi:10.1186/s13063-023-07509-4)
Supplement: Supplementary file 1 — Additional file 1. Full study protocol version 4.1. [file 13063_2023_7509_MOESM1_ESM.pdf]

Department of Clinical Neuroscience

Protocol HD RCT

Long title of the trial	In-home decluttering augmentation of group cognitive-behavior therapy for hoarding disorder: The Joining Forces randomized controlled trial
Short title of trial	HD RCT
Version and date of protocol	Version 4.1, 2023-04-27
Sponsor	Stockholm Healthcare Services, Psykiatri Sydväst.
Funder (s)	Forte (grant number 2019-01637)
Trial registration number	ClinicalTrials.gov: NCT04712474
Sites	OCD-programmet<br>Psykiatri Sydväst, Stockholm Healthcare Services<br>Huddinge sjukhusområde M46,<br>Medicingatan plan 4, 141 86 Stockholm, Sweden<br><br>Samlarteamet<br>Medborgarplatsen 21<br>118 72 Stockholm, Sweden
Principal investigator:	Dr Volen Ivanov: volen.ivanov@ki.se<br>Department of Clinical Neuroscience (CNS), K8, CPF Rück M48, Karolinska Universitetssjukhuset Huddinge, 141 86, Stockholm, Sweden
Sponsor representative:	Mats J. Olsson, professor and Head of Department<br>Karolinska Institutet<br>Department of Clinical Neuroscience (CNS),<br>K8 Klinisk neurovetenskap, 171 77 Stockholm<br>mats.j.olsson@ki.se<br>+46-(0)8-524 824 64

Version history

Version number	Version date	Reason for Change
1.0	2020-10-26	First version
2.0	2021-06-30	Change inclusion criteria of geographical uptake, the need of a means approval from the social services, add a question regarding preferences toward randomization results and reduce monitoring to random sample instead of monitoring of all case report forms.
3.0	2021-11-17	Change of platform to report interest in participating and add an anonymous survey regarding the coronavirus pandemic.
4.0	2022-11-11	Addition of a process evaluation.
4.1	2023-04-27	Minor changes of typing errors and adding information needed before submission to scientific journal.

Signature

Protocol version number: 4.1

Volen Ivanov, Principal Investigator (PI)

Date: 2023-04-27

Signature:

VOLEN IVANOV, MED DR

TABLE OF CONTENTS

1 Trial personnel 5
2 Summary 7
3 Introduction 9
3.1 Background 9
3.2 Management of Hoarding Disorder 9
4 Objectives 11
4.1 Primary and secondary objectives 11
4.2 Research questions and hypotheses 11
5 Project description 12
5.1 Design 12
5.1 Study sites 12
5.1.1 Stockholm 12
5.2 Inclusion and exclusion criteria 13
5.2.1 Inclusion criteria 13
5.2.2 Exclusion criteria 13
6 Procedure 14
6.1 Recruitment 14
6.1.1 Recruitment rate 14
6.1.2 Screening and recruitment procedures 14
6.2 Cognitive-behavioral Therapy 15
6.2.1 Coordination of cbt and in-home decluttering 16
6.3 Randomization, enrolment, masking 18
6.3.1 In-home decluttering 18
6.3.2 waitlist condition 19
6.4 Concomitant interventions 19
6.5 caregivers and relatives 19
6.6 Qualitative Data Collection 20
7 Measures and measurement points 20
7.1 End of trial 24
7.2 Participant withdrawal from trial 24
7.3 Noncompliance and discontinuation of trial 24
7.3.1 Noncompliance 24

7.3.2 Discontinuation of trial ... 25
7.4 Quality control ... 25
8 Data management ... 25
9 Statistical analysis ... 25
9.1 Sample size calculation and statistical analysis ... 25
9.2 Cost-effectiveness analysis ... 26
9.3 The process evaluation ... 26
10 Recording and reporting of adverse events ... 27
11 Public and patient involvement ... 27
12 Data sharing ... 28
13 Ethical considerations ... 28
13.1 The Covid-19 pandemic ... 29
14 Implications ... 29
15 Finance ... 29
16 Publications ... 29
17 Clinical infrastructure ... 29
18 References ... 31

1 TRIAL PERSONNEL

Principal Investigator (PI):	Dr Volen Ivanov<br>Karolinska Institutet<br>Department of Clinical Neuroscience (CNS), K8,<br>CPF Rück, 171 77 Stockholm<br>volen.ivanov@ki.se<br>+46 725 19 06 73
Cooperative representatives:	Lina Martinsson, head of operations<br>Psykiatri Sydväst, Stockholm Healthcare Services<br>Huddinge sjukhusområde M58,<br>Medicingatan 8, plan 5, 141 86 Stockholm<br>lina.martinsson@sll.se<br>+46 8 585 865 35<br><br>Veronica Starck, head of operations<br>Enkede-Årsta-Vantörs stadsdelsförvaltning.<br>Box 81, 121 22 Johanneshov
Co-investigators:	Professor Christian Rück<br>Karolinska Institutet<br>Department of Clinical Neuroscience (CNS) K8,<br>CPF Rück, 171 77 Stockholm<br>christian.ruck@ki.se<br>+46 70 484 33 92<br><br>Professor David Mataix-Cols<br>Karolinska Institutet<br>Department of Clinical Neuroscience (CNS), K8,<br>Gävlegatan 22, Plan 8, 113 30 Stockholm, Sweden<br>david.mataix.cols@ki.se<br>+46 72-398 68 89<br><br>Dr Lorena Fernández de la Cruz<br>Karolinska Institutet<br>Department of Clinical Neuroscience (CNS), K8,<br>Gävlegatan 22, Plan 8, 113 30 Stockholm, Sweden<br>lorena.fernandez.de.la.cruz@ki.se<br>+46 76 847 79 99<br><br>Dr Erik Andersson<br>Karolinska Institutet<br>Department of Clinical Neuroscience (CNS), K8,

Nobels Väg 9, 171 77 Stockholm
erik.m.andersson@ki.se
+46 73-671 63 35

Professor Randy Frost
Smith College
Bass Hall 307, 10 Elm Street, Northampton, Massachusetts 01063
rfrost@smith.edu
+1 413-585-3911

Dr Carolyn Rodriguez
Stanford University
Department: Psych/Public Mental Health & Population Sciences
401 Quarry Road, Stanford, California 94305
carolynrodriguez@stanford.edu
+ 1 (650) 723-6158

Sofia Jägholm
Karolinska Institutet
Department of Clinical Neuroscience (CNS), K8, CPF Rück, 171 77 Stockholm
sofia.jagholm@ki.se
+46 708 10 20 64

Patient representative:

Elin Olaisson
OCD-föreningen Stockholm
elin.olaisson@ocdstockholm.se
+46 8-669 30 39

2 SUMMARY

Title:	In-home decluttering augmentation of group cognitive-behavior therapy for hoarding disorder: The Joining Forces randomized controlled trial.
Short title:	In-home decluttering augmentation of group CBT for HD.
Objectives:	Primary objective: To determine the clinical efficacy of *in-home decluttering* augmentation of group CBT for reducing hoarding severity in adults with HD.<br><br>Secondary objectives: To assess the cost-effectiveness of *in-home decluttering*, from a social services provider perspective.
Type of trial:	Single-blind parallel-group randomized controlled superiority trial.
Trial design and methods:	Participants who are eligible and have consented to participate in the trial will receive protocol-driven group CBT for HD for 12 weeks. After group CBT, participants are immediately randomized to one of two trial arms. In the experimental arm, participants receive 10 weeks of in-home decluttering. In the waitlist arm, participants receive no intervention but fill out questionnaires with identical procedures as in the intervention group.<br><br>Participants will complete outcome measures at pre-CBT, mid-CBT (week 6), baseline (i.e. immediately after receiving CBT and before in-home decluttering), twice during in-home decluttering (week 4 and 7), post-treatment (week 10 of in-home decluttering), and 3-, 6-, and 12-months post-treatment. Two primary outcome variables will be used in this study; the total score on the Saving Inventory- Revised (SI-R) and the total score on the clinician-reported Clutter Image Rating (CIR). The primary endpoint is post-treatment (i.e. week 10 of in-home decluttering or waitlist).<br><br>Secondary outcomes include participants' cognitions about discarding (SCI), relationship between self and items (RSI), measures of clutter (CIR), squalor (HEI), impairment of daily functioning (ADL-H), severity and improvement (CGI), adverse events, and treatment satisfaction (CSQ-8). Relatives of trial participants will rate levels burden and accommodation with the Family Impact Scale for Hoarding Disorder (FISH) and the Caregiver Burden Inventory (CBI), respectively. The Trimbos Questionnaire for Costs associated with Psychiatric Illness will be used to assess healthcare and societal resource use. Follow-up assessments will be conducted at the clinic or via videoconferencing.

Participant time in trial:	Approximately 17-22 months.
Total trial duration:	Approximately 42 months (from first participant enrolled to last participant at follow-up).
Planned trial sites:	Group CBT will be administered at one psychiatric unit (OCD-programmet in Huddinge, Stockholm). The in-home decluttering intervention will be delivered by a specialized team from a social services unit (Samlarteamet, Stockholm). Assessment procedures will be a combination of assessments at the psychiatric unit and in each participant's home.
Sample:	90 participants.
Brief eligibility criteria:	Eligible participants will be aged 18 years or older who fulfill criteria for a diagnosis of HD according to DSM-5. Exclusion criteria include previous or current CBT for HD, unable or unwilling to allow study staff into their home, animal hoarding, severe squalor or home conditions that would put the personnel at risk during home visits, a diagnosis of organic brain disorder, intellectual disability or psychosis, among others. Full details are presented in section 5.2.
Statistical methodology and analysis:	Data will be analyzed according to the intention-to-treat principle. The primary outcome measures will be analyzed using linear mixed effects models. Secondary outcomes will be analyzed using analogous methods to the primary outcomes.

3 INTRODUCTION

3.1 BACKGROUND

Hoarding disorder (HD), included in 2013 as a separate mental disorder in the latest edition of the Diagnostic and Statistical Manual of Mental Disorders (DSM–5) [1] and more recently in the International Classification of Diseases (ICD-11) [2], occurs in approximately 2.5% of the population [3], is difficult to treat, and requires a coordinated response from the community [4]. Individuals who hoard experience significant difficulties discarding or parting with possessions and intense emotional distress when having to discard them [1]. These individuals often also have strong urges to acquire items. Together, these symptoms result in the accumulation of large amounts of items that are kept in a disorganized manner (i.e., clutter) at home, to the degree that normal use of the space is difficult, causing significant impairment in function [1]. For instance, the homes of individuals with HD can be so cluttered that cooking, bathing, and finding important things becomes difficult or impossible [5]. Moreover, study prevalence rates of squalor (i.e., the accumulation of dirt, grime, human or animal waste or rotting food) across three diverse agencies suggest that squalor is common in hoarded homes (35–72%) [6], but far from ubiquitous. The consequences of hoarding include health code violations and an increased risk for eviction and homelessness [7], and, in some rare instances, even serious injury or death caused by the fire hazards or "clutter avalanches" the individual is buried under [8]. Furthermore, those who care for or live with people with HD also experience functional impairment and emotional distress. Notably, the level of self-reported care burden is comparable to that observed among care givers to individuals with dementia [9].

HD not only affects the individuals and families, but also constitutes a severe public health burden. The extreme accumulation of possessions increases the risk for fire hazards, especially if flammable materials (e.g., newspapers) are piled near stoves and furnaces, putting both the individual and neighbors at risk [8]. Community agencies then must intervene to protect the individual and those living nearby. As a result of the impact on society, a wide range of community organizations are burdened with the costs for firefighting, sanitation, and eviction and spend tremendous time and financial resources on HD, including clean-outs and provision of housing [8].

3.2 MANAGEMENT OF HOARDING DISORDER

Currently, behavioral approaches are the most commonly used to treat HD, and a variety of interventions (both in an individual and group format) based on cognitive-behavioral therapy (CBT) and designed specifically for individuals with HD have been developed and tested over the last decade [10-12]. CBT for HD typically includes psychoeducation about the disorder, motivation enhancement, learning skills to resist triggers to acquire (e.g., during trips to flea markets), cognitive exercises, and imagined and actual discarding of objects. Both group and individual treatment approaches have been examined for efficacy in HD and a meta-analysis showed large effect sizes for CBT for HD interventions, regardless of treatment format (group vs. individual). However, in the vast majority of individuals with HD, impairing symptoms remain after treatment (up to 75% when using a conservative estimate of hoarding symptom change), especially with respect to the level of clutter in the home [13]. The majority of people with HD thus, live in cluttered homes that impair their functioning even after a full course of

evidence-based treatment, putting them at continued risk for adverse consequences such as eviction and fire hazards. A meta-analysis examining predictors of treatment outcome after CBT for HD, revealed that the number of home visits predicted better outcome [13]. This finding suggests a need to complement CBT with home-based assistance in order to target the residual impairing clutter.

In Sweden, individuals with HD are among the group of people with mental disorders who receive care and support from both health care and the social services. These individuals are those with psychiatric disabilities, defined as a lasting (>2 years) incapacity to manage everyday life [14]. People with HD who receive support from the social services mainly live in supported accommodation homes with varying levels of outreach housing support [15]. The municipalities (Swedish: *kommuner*) are responsible for providing this support. Residents who receive outreach housing support live in an apartment or house that they rent or own and are provided support by a social worker (Swedish: *boendestödjare*) up to three times a day by the municipal outreach housing services. The tasks of outreach housing support staff usually include assisting clients with their daily self-care activities, reducing social isolation, and helping clients manage and coordinate their contacts with health care and authorities. The staff frequently encounter individuals with HD and are required to provide them with strategies for coping with the accumulation of clutter and managing everyday activities. Although this support frequently spans over months and sometimes years and is thus costly for the municipalities, providing individuals with HD with effective support has proven to be an immense challenge for the social services in Sweden. The staff do not have access to any structured, evidence-based model for support adapted for HD and instead have to resort to unstructured supportive interventions with unknown effects. Professionals in social services recognize these challenges and consequently report the lack of clinical management tools and training for HD along with formal collaboration with health care services as the barriers that prevent them from providing effective care for people with HD [16].

Members of our team have previously implemented manualized group-based CBT for HD [17] in mental health care and the treatment is now regularly provided at a specialized psychiatry unit in Stockholm (*OCD-programmet*, Stockholm Health Care Services). Our research group has shown that the treatment is feasible and associated with a low drop-out rate [18]. Moreover, although this treatment is effective with outcomes in line with those typically reported in other trials [13], the effects on clutter remain modest with residual clutter impairing the patients after treatment.

Despite the impairment and negative repercussions of clutter in individuals with HD, the community is frequently faced with the challenge that individuals with HD are often hesitant to seek treatment, possibly due to the generally low levels of insight as well as to the stigma associated with the disorder. Thus, identifying what aspects and types of current treatments are most acceptable is a crucial part of treatment development. In order to understand the perspective of the individual with HD better, members from the project team conducted an online survey study of treatment acceptability in participants (n=203) with clinically meaningful hoarding symptoms. Participants reported that personalization of care (e.g., individual sessions customized to the individual's unique in-home needs) and accountability

made treatments most acceptable [19]. Based on these findings and those in a meta-analysis reporting that a greater number of home sessions is associated with better clinical outcomes [13], members of the project team added a personalized, in-home declutter component to evidence-based treatment for HD. The pilot results from a study conducted in New York, showed that adding this brief in-home decluttering component decreased the overall clutter level in the home and may have benefited some individuals who might have not otherwise had clutter level reduction. Study participants also reported improvement in activities of daily living and the procedure was feasible and well tolerated [20].

In parallel with the development and implementation of psychological treatment for HD, since 2013, our research group has been collaborating with the social services in Stockholm. As part of this collaboration, our research group co-founded a team within the social services (named *Samlarteamet*) which specializes on providing in-home decluttering to clients with cluttered homes. The team was formed in 2017 and has since been developing and testing a novel model of care for individuals with HD. This model is based on the social services' traditional format of outreach housing support, but differs from it significantly by including several essential elements of CBT for HD. These elements include goal and agenda setting, organizational skills training, exposure-based exercises to target emotional avoidance, and homework assignments. Currently, the team consists of 5 social workers who devote part of their time to working with clients with HD.

In summary, CBT is efficacious but most patients need additional support to reduce clutter and increase overall function. In-home decluttering by social services is a promising model for achieving this, but whether it can augment CBT in reducing hoarding symptoms severity and clutter in particular, has not yet been tested.

4 OBJECTIVES

4.1 PRIMARY AND SECONDARY OBJECTIVES

Building on previous work showing the feasibility and preliminary efficacy of adding in-home decluttering sessions to CBT, we plan to test the efficacy of augmenting group CBT for HD with 10 subsequent home visits, delivered over 10 weeks, in a randomized waitlist-controlled trial (RCT). Our secondary aim is to explore the cost-effectiveness of adding this component to CBT and to evaluate the processes surrounding in-home decluttering.

4.2 RESEARCH QUESTIONS AND HYPOTHESES

1) Does adding a subsequent, personalized, in-home decluttering component to CBT augment its efficacy, compared to CBT (i.e. waitlist)? Specifically, does it result in:
    a) a decrease in self-reported symptoms of HD (measured with the self-reported Saving Inventory-Revised)?
    b) a decrease in levels of clutter (measured by blind raters using the Clutter Image Rating)?
    c) an increase in daily self-care activities (measured with the self-reported Activities of Daily Living – Hoarding)?

d) a decrease in caregiver burden (measured by the Caregiver Burden Inventory) and levels of accommodation (measured by the Family Impact Scale for Hoarding) among relatives of participants in the trial.

2) What is the incremental cost-effectiveness ratio of in-home de-cluttering?

3) What are the mechanisms of impact and the contextual factors that might have affected the in-home decluttering intervention and its outcomes?

We hypothesize that adding a personalized, in-home decluttering component will augment group CBT by further reducing self-reported hoarding symptoms, reducing clutter levels, improving activities of daily living as well as reducing caregiver burden and decreasing accommodation to hoarding behaviors among relatives. We will also estimate the treatment costs and plot a cost-effectiveness acceptability curve of the intervention using different willingness-to-pay scenarios. We will take three different scenarios; (A) social services provider perspective, (B) social services sector perspective. As a second health-economic analysis, we will estimate the cost-effectiveness of the intervention expanding the costs to also include a full societal perspective (i.e. including health care costs and costs associated with productivity loss).

5 PROJECT DESCRIPTION

5.1 DESIGN

This study is a single-blind, parallel-group, superiority trial. All study participants will first undergo protocol-driven group CBT for 12 weeks [17] and within 10 days after CBT, be randomly assigned to 10 weeks of in-home decluttering or waitlist in a 1:1 ratio, without restriction. To prevent a potential selection bias related to the randomization procedure, an external party, the Karolinska Trial Alliance (KTA, www.karolinskatrialalliance.se), not involved in the inclusion process, will be in charge of the randomization by using a true number service (www.random.org). Allocation concealment will be ensured through randomization after the decision to include each participant has been made. Immediately after randomization, participants will receive information about which condition they have been allocated to. Participants randomized to waitlist will be offered in-home decluttering after an approximate 10-week delay, i.e. after the primary endpoint.

5.1 STUDY SITES

5.1.1 STOCKHOLM

This trial is a collaboration between the Stockholm Healthcare Services (SHS), Stockholm's social services and Karolinska Institutet. The group CBT will be administered at one psychiatric unit within SHS (*OCD-programmet* in Huddinge, Stockholm) and the participants will be considered as patients at the clinic. The in-home decluttering will be delivered by a unit within the Stockholm Social Services and the actual intervention will take place in each participant's home. Both trial sites are located in Stockholm, which enables coordination of the two parts of the treatment.

5.2 INCLUSION AND EXCLUSION CRITERIA

5.2.1 INCLUSION CRITERIA

1) Age 18 years or older.
2) Hoarding disorder as primary psychiatric condition.
    - Confirmed by the assessor at the psychiatric assessment.
3) Willing and able to understand and complete consent and study procedures.
4) Living in the municipality of Stockholm OR
    living within a 1-hour commute by public transport from the social services office delivering the in-home decluttering intervention and have means approval of specialized in-home decluttering from their social service unit

5.2.2 EXCLUSION CRITERIA

1) Concurrent CBT or having received CBT for HD during the last two years, for a minimum of 8 sessions including active strategies for reducing acquisition and practice of discarding with a qualified therapist *or* 8 previous in-home decluttering sessions with a qualified social worker.
    - Confirmed by the participant or assessor at the telephone screening or psychiatric assessment.
2) Unable or unwilling to allow study staff into home for home assessment.
    - Confirmed by the participant or assessor at the telephone screening or psychiatric assessment.
3) Animal hoarding or squalid (i.e. extremely unhygienic) home conditions that are deemed to put the personnel at risk during the in-home decluttering.
    - Confirmed by the assessors at the home assessment.
4) A diagnosis of organic brain disorder, intellectual disability, psychosis, anorexia nervosa, alcohol/substance dependence or abuse or bipolar disorder without stable medication or with symptoms during the last 6 months.
    - Confirmed by the assessor at the psychiatric assessment, with help of information from the participant and the MINI interview.
5) Major medical or neurological conditions that would prevent completing assigned behavioral practice tasks.
    - Confirmed by the participant or assessor at the telephone screening or psychiatric assessment.
6) Immediate risk to self or others, requiring urgent medical attention, such as high suicidality risk.
    - Confirmed by the participant or assessor at the telephone screening or psychiatric assessment.
7) Participant not able to read and communicate in Swedish.
    - Confirmed by the participant or assessor at the telephone screening or psychiatric assessment.
8) Currently at high risk for eviction (for instance having received a "*rättelseanmaning*" by the housing company or Swedish court).

- Confirmed by the assessor at the telephone screening, psychiatric assessment or home assessment.

9) Potential participant lives in the same household as an already included participant.

6 PROCEDURE

6.1 RECRUITMENT

6.1.1 RECRUITMENT RATE

Recruitment is planned to be conducted within a 36-month period. Based on our calculations, we need to randomize 90 participants (see power calculations under section 9.1). Although previous treatment trials have shown high non-completion rates, (around 25-30%) [11, 12], the drop-out rates have been considerably lower in our clinic [18]. Based on those, we estimate a drop-out rate of around 15%. However, due to the risk of drop-outs before randomization, during or immediately after CBT, we aim to recruit around 100 participants to the first phase of the study (group CBT).

Based on our prior experience, randomization of 90 participants during 2-3 years is realistic. The two study sites have a regular flow of approximately 30 clients with HD per year. We will also recruit through the Stockholm Task Force, other health- and social services units, as well as via online self-referral. The Stockholm Task Force consists of 20-30 members (from academia, psychiatric- and social services, housing- and patient organizations and the Enforcement authority) who regularly meet to discuss hoarding related questions and commitments.

6.1.2 SCREENING AND RECRUITMENT PROCEDURES

We plan to advertise the trial to clinics and social services across Stockholm County, patient organizations, as well as directly to the public via our website and social media. If needed, we might publish paid advertisements in traditional and social media. Participants can be referred to the trial in three different ways: (1) via self-referral (through the trial website), (2) via clinical referral to the mental health clinic *OCD-programmet* (through the medical record system TakeCare), and (3) via the social services units in Stockholm municipality or referral from the Stockholm Task Force.

All referred potential participants will initially be contacted via telephone by a member of the research team (*telephone screening*) or, in some cases, approached face-to-face at the clinic. The main purpose of this contact is to perform a preliminary screening of eligibility, which includes asking brief questions about hoarding symptoms using the Hoarding Rating Scale – Interview (HRS-I) [21], and each inclusion/exclusion criterion (see section 5.2 for full information and Figure 1. If preliminarily eligible and interested in participating in the trial, the participants will be booked for the next step (*psychiatric assessment*), carried out by a psychologist or psychiatrist at the psychiatric clinic *OCD-programmet*, Psykiatri Sydväst, Stockholm Health Care Services. The aim of this visit(s) is to a) verify that diagnostic criteria for HD (except clutter) are met and b) assess HD severity level. See Table 1 for more information about the assessment. During the psychiatric assessment, the assessor also gives

careful information about the study protocol and outlines the time commitment associated with participation, as well as answers any questions from the participants.

Some of the exclusion criteria, such as the presence of squalor in the home, will be assessed during the final step (*home assessment*) of the recruitment process. The home assessment is carried out by one psychologist and one social worker and the aims are to confirm the HD diagnosis and determine whether delivering in-home decluttering would be feasible. Presence of squalor is measured with the clinician-reported Home Environment Index [22] and possible risks are assessed with the risk section in the Structured Interview for Hoarding Disorder [23]. Levels of clutter will be assessed by the assessor using the Clutter Image Rating [24]. During the home assessment, the assessor will explain the aims, methods, benefits, and hazards of participating. Further, s/he will explain that the participants are under no obligation to enter the trial and that they can withdraw at any time, without having to give a reason. If all inclusion criteria, and no exclusion criteria, are met, the participant signs the informed consent during the home assessment and is preliminary included in the trial. Before the participants are fully included, they need means approval from the social service unit to receive the in-home decluttering in the trial. Study personnel will help the participant with the means application after receiving the informed consent. No deviations or waivers to the eligibility criteria are allowed. If the assessor is uncertain whether a potential participant is eligible, the PI will be informed and have the final decision.

When the consent form has been signed by the participant and all baseline measures have been completed, the participant will be assigned a trial ID and enrolled in the study. The signed consent form will be retained in a locked file cabinet. A log will be kept matching screening IDs with trial IDs. Since a full treatment group (5-9 participants) needs to be recruited before CBT starts, participants will be placed on a waiting list after being included until a sufficient number of participants have been included.

Finally, if the participant is not included in the trial (regardless of the reason), but still requires clinical attention, our team will initiate a referral to other suitable services, whenever possible.

6.2 COGNITIVE-BEHAVIORAL THERAPY

All included study participants will first receive group CBT for HD, delivered at a mental health care site specializing in HD (*OCD-programmet*, Psykiatri Sydväst, Stockholm Health Care Services). We will primarily aim to include 8 participants in each group, but the number of participants can vary from 5 to 9.

Group CBT comprises 12, 2-hour sessions. Each session will be facilitated by two psychologists, of which at least one has previous experience in treating HD. Group CBT will follow the Swedish version of a published manual based on the current psychological model for HD [17]. The original manual includes 16 weekly, 1.5-hour sessions. However, in the current trial, the treatment will be delivered in 12, weekly 2-hour sessions. All components from the original manual will thus be delivered for the same amount of time (24 hours), albeit during a shorter time frame. The shortening of the time frame is due to the long nature of the combined intervention (CBT + in-home decluttering; see 6.4.1) and thus the risk of treatment

fatigue among the participants. Evidence from one study suggests that the efficacy of this shorter form of CBT (i.e. 12 sessions) is similar to the efficacy of longer group- and individual CBT treatments for HD [25].

The manual includes psychoeducation about CBT and HD, goal-setting, executive skills training, cognitive restructuring, mindfulness-based skills to accept and tolerate negative emotions, motivation enhancement, learning how to combine these components, and relapse prevention. Group sessions start with a review of the homework assignment from the previous week, followed by a review and exercises regarding a specific hoarding-related topic. The efficacy of the treatment based on this manual has been tested in a large RCT [12] and our research group has tested the feasibility of delivering the treatment in the Swedish healthcare context [18].

The group CBT will be facilitated by two psychologists, of which at least one with previous experience in conducting CBT for HD. Adherence to the treatment protocol will be monitored: the treating psychologist will record which treatment components were administered by using the checklist at the end of each session.

6.2.1 COORDINATION OF CBT AND IN-HOME DECLUTTERING

After the last group CBT session, an additional individual session will take place, during which the study participant will meet with 1 of the group facilitators (psychologists) and the 2 social workers who will be providing the in-home decluttering service. During this meeting, the study participant's CBT treatment and progress will be jointly reviewed and a detailed plan for the upcoming home-visits be made.

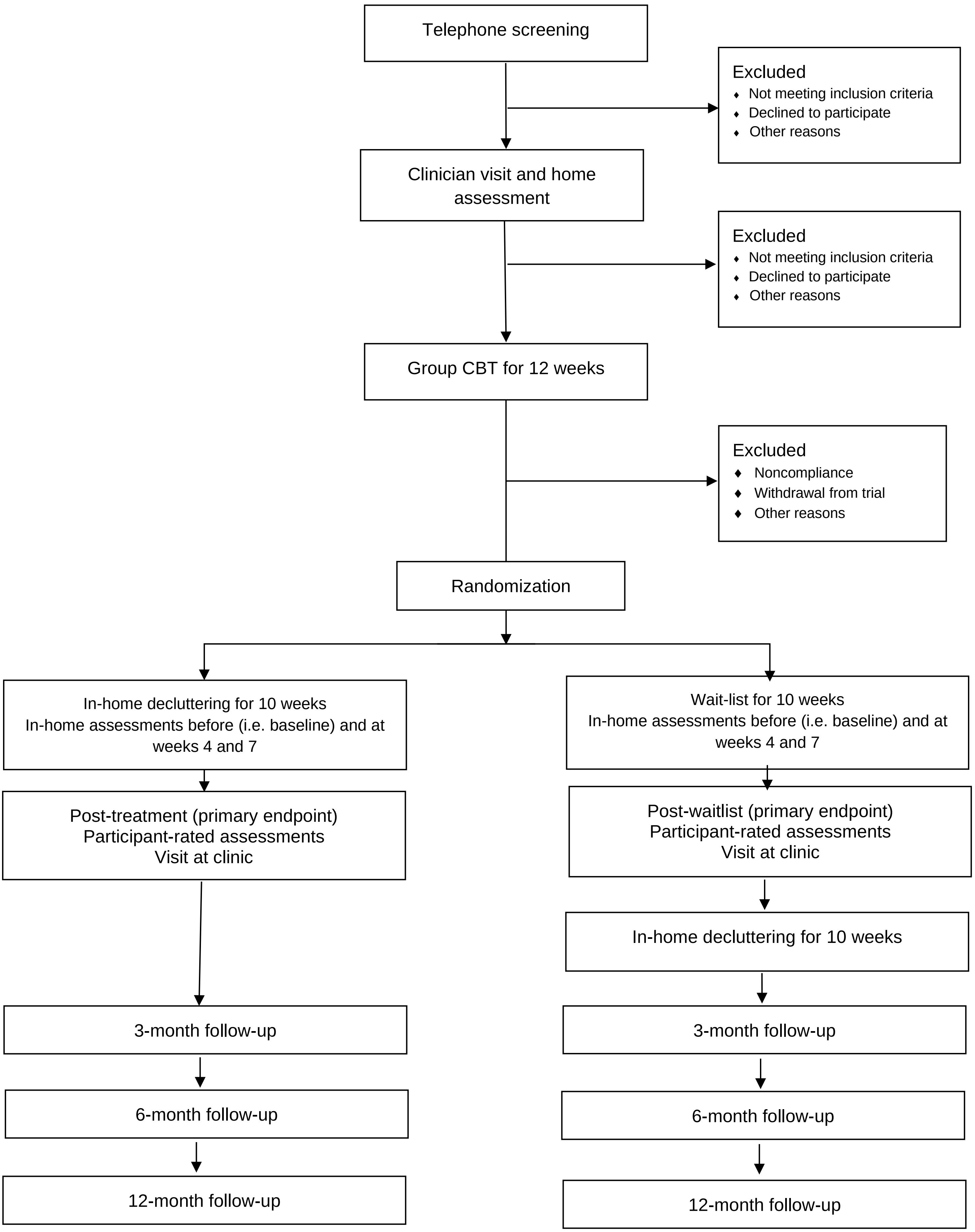

Figure 1. CONSORT diagram over patient flow.

6.3 RANDOMIZATION, ENROLMENT, MASKING

After group CBT, study participants will be randomized at a 1:1 ratio to either in-home decluttering or waitlist. See Figure 1 for overview of the patient flow. The randomization sequence will be generated before randomization of the first participant, using masked block randomization by an independent entity which will not be involved in the trial (Karolinska Trial Alliance; https://karolinskatrialalliance.se). Randomly varying block sizes will be generated using a computer random number generator. Social workers delivering the in-home decluttering intervention will be randomly assigned (in pairs) to each participant. Participants will be informed of group assignment by the project coordinator after the completion of CBT.

Assessors conducting post- and follow-up assessments will be blind to condition allocation until after the primary endpoint (post in-home decluttering). The outcome measures are identical for both groups, ensuring that the assessors remain blind. At each follow-up assessment, participants will be reminded by their assessor to not reveal their arm allocation. To measure blinding integrity, all visits will be audio recorded and all assessors will register whether the participants inadvertently reveal their group allocation, and subsequently guess each participant's treatment allocation at each assessment point and motivate their choice. If the treatment allocation is accidently revealed, that very part will be cut out of the audio recording. A new blind assessor will then listen to the recording and conduct the rating that will be used in the trial.

6.3.1 IN-HOME DECLUTTERING

The in-home decluttering begins after randomization and will be delivered by social workers (Swedish: *boendestödjare*). It includes 10, 1.5-hour home-visits over the course of 10 weeks. These home visits are personalized, agenda driven, goal-oriented, structured, and focused on decluttering. They include 1) a brief check-in, 2) guided unclutter time (including motivational enhancement, cognitive exercises, and exposure to aversive emotions), 3) a reflective period in which participants share their thoughts and objectives for the coming week, and 4) homework assignments. The content in the home-visits thus mirrors the format that the study participants are familiarized with through the CBT. However, the purpose of the home-visits is to focus on practical management of clutter (i.e., removing and reorganizing) with a clear emphasis on improving the participants' ability to perform daily activities in their home (e.g., clearing the kitchen in order to be able to cook and the sofa to be able to invite visitors).

Prior to the start of the decluttering intervention, all staff (social workers) who deliver in-home decluttering will read a treatment manual, specifically developed for this trial and undergo a one-day workshop delivered by an expert in HD and members from the social service team specialized in decluttering (*Samlarteamet)*. The content of this workshop will focus on managing HD, strategies for delivering in-home decluttering and the study procedures. Members of staff who have worked with clients with hoarding for longer than 6 months will not be offered the workshop but will receive a thorough review of the study procedures. During the trial, the staff will receive regular supervision by experts in HD (supervisors). Moreover,

adherence to the in-home decluttering protocol will be monitored using a checklist of administered components after each session, filled out by one of the social workers.

6.3.2 WAITLIST CONDITION
Participants assigned to waitlist will receive no in-home decluttering for 10 weeks after group CBT. During this time period, an assessor from the study will visit the participants every third week (corresponding to treatment weeks 4 and 7 in the treatment group) in order to complete the pertinent assessment measures and have photographs taken the home for blinded CIR-ratings. Audio from all home visits will be recorded and monitored by supervisors in order to ensure that no treatment interventions were given during these visits. While on waitlist, participants will also be encouraged to contact the psychiatric clinic in case of a psychiatric emergency (e.g., increased suicidal ideation).

After 10 weeks (primary endpoint), participants on the waitlist will be crossed over and offered the same intervention as the active group. In-home sessions may however in some instances be delayed for up to 5 weeks (after the initial 10-week waitlist) for some participants. This may occur if the end of the waitlist period coincides with the summer vacation for the social workers. In this instance, participants on waitlist will also be given the option to commence the in-home intervention immediately after waitlist, pause it up to 5 weeks, and resume the final session after the summer.

6.4 CONCOMITANT INTERVENTIONS
Participants are free to continue any medication during the trial. In the case of a comorbid bipolar disorder treatment for the condition is required to have been stable (type of drug and dosage) for the six consecutive months prior to the inclusion in the trial. Further, the participants are asked to not start any parallel/new psychological treatment (of any type) for the indication of HD until after the 3-month follow-up. There is no well-established pharmacological medical treatment for hoarding symptoms. Therefore, the participants can change psychiatric medication in accordance to standard practice recommended by their usual treating clinician for the duration of the trial. Potential medication changes will be noted at the follow-up assessments.

Concomitant interventions from the social services, such as outreach housing support (Swedish: *boendestöd*), will be allowed as long as this intervention does not focus on the participants' hoarding difficulties or clutter. This will be confirmed and coordinated by the study personnel after inclusion, either through a meeting or by phone.

6.5 CAREGIVERS AND RELATIVES
The level of care burden experienced by relatives of people with HD is high [9] and it is therefore important to investigate if and how family members are affected by the interventions in this trial. Before enrolling in the trial, each participant will be asked if they have a relative (or other significant person) that may be contacted by the researchers. If the participant consents to this, the relative will be contacted and asked to fill out questionnaires of having a relative in treatment for HD at the beginning, during and at the end of the treatment. The relative will be informed that participation is voluntary. They will receive information about the study and the questionnaires by mail.

6.6 QUALITATIVE DATA COLLECTION

In order to get a better understanding of the mechanisms of impact and the contextual factors that might have affected the implementation process and its outcome we will collect qualitative data. The qualitative data will consist of semi structured interviews with, 1) the participants randomized to the in-home decluttering intervention (n=15-20), 2) social workers (Swedish: "boendestödjare") who will deliver the intervention (n=4-8) or in other ways are involved in the trial (assistants and managers. The semi-structured interview questionnaires have been specifically developed for this trial and cover the following topics:

Personnel/social workers:

- How they learned about the trial
- Their experience of delivering the intervention and following the manual
- Knowledge needed to be able to deliver the intervention

Participants:

- How they learned about the trial and why they applied
- Their experience of in-home decluttering and their contact with the personnel delivering the intervention
- Feedback regarding the content of the intervention

The participants will be purposively sampled (e.g. different ages and sexes and varying levels of insight) with the intention to obtain varying views of in-home decluttering, ensuring that different perspectives are voiced. We anticipate that this sampling strategy will result in sufficient heterogeneity to provide examples of both poor and good engagement and delivery, allowing us to identify barriers and facilitators to implementation and to generate hypotheses about factors that may be associated with differing outcomes for persons with HD. The social workers on the other hand will be asked to participate in interviews at different time points to better understand the process of the implementation and experiences at different parts of the trial. The interviews with the social workers will be conducted early in the trial and near the end of the recruitment of all participants. All social workers involved in the trial will be asked to participate. The health care personnel will be asked about their experience of the coordination of the two interventions (6.2.1) at the end of the trial.

7 MEASURES AND MEASUREMENT POINTS

The diagnosis of HD will be assessed using the **Structured Interview for Hoarding Disorder** (SIHD) [23], a semi-structured interview containing the DSM-5 diagnostic criteria for HD and its specifiers, as well as potential safety risks in the context of HD. It will be used both at pre-CBT and to assess remission status at primary endpoint and follow-ups. To assess psychiatric comorbidities and guide clinical judgment at baseline, the **Mini International Neuropsychiatric Interview** [26] and a short diagnostic interview, the **OCD-RD**, originally based on the Structured Clinical Interview (SCID) for DSM-5 [27], will be administered. Before the psychiatric assessment screening for symptoms of depression and substance use will be administrated using **Montgomery-Asberg Depression Scale** (MADRS-S) [28], **Alcohol**

User Disorders Identification Test (AUDIT)[29] and **Drug User Disorders Identification Test** (DUDIT)[30].

Assessments will be conducted at pre-CBT, mid-CBT, post-CBT/baseline, in-home decluttering week 4, in-home decluttering week 7, post in-home decluttering, and 3-, 6-, and 12-month follow-up. **Table 1** lists all assessments at the different time points. The primary endpoint is post in-home-decluttering (week 10). The assessment procedure at the primary endpoint consists of 2 parts; a) administration of self-reported questionnaires immediately after the last home visit and b) a visit at the clinic or, if needed, video meeting or a separate home visit by psychologist/psychiatrist, no more than 2 weeks after the last home visit or week on waitlist. The assessment at the clinic is conducted by a psychologist or psychiatrist blinded to the participant's trial arm allocation.

The primary outcome measures are the **Saving Inventory-Revised** (SI-R) [31] and the **Clutter Image Rating** (CIR) [24]. The SI-R will be administered pre-CBT, at the sixth session of CBT (mid-CBT), post-CBT (baseline), mid-in-home decluttering (week 4 and 7), post in-home decluttering (week 10; primary endpoint) and at 3-, 6- and 12-month follow-up. The CIR will first be used by the assessors during the home assessment. Further on, throughout the trial, the CIR will be used as both a self-rated measure and a measure rated by blind assessors. The assessors, and subsequently, the social workers, will take photographs of the participants' homes, which will be rated with the CIR by blind assessors not involved in any parts of the treatment in the trial. This procedure will be carried out prior to the first in-home session in the in-home decluttering group and during the first week in the waitlist group (baseline), as well as at weeks 4 and 7 and at post in-home decluttering (week 10). At every assessment point, the participants will also be asked to rate the level of clutter in their home using the CIR. No home visits will be carried out at the remaining assessment points (3-, 6- and 12-month follow-ups), and instead, the CIR will be rated by the participants and rated by blind assessors (if participants are willing and able to provide photographs of their home).

Secondary blinded clinician-administered outcome measures include the **Clinical Global Impression Scale** (CGI) [32], a brief assessment of the clinician's view of the patient's global functioning. It consists of measures of the severity of illness (CGI-S) and global improvement after treatment (CGI-I). Both measures will account for HD-related symptoms only.

Participants will self-rate their cognitions about discarding their possessions using the self-report measure the **Saving Cognitions Inventory (SCI)** [33] and their level of object attachment by using the visual measurement **Relationship between Self and Items (RSI)** [34], which is an adaption of the measurement Inclusion of Other in Self scale [35]. The **EuroQol-5D** [36], which provides a single index value that will be used in both clinical and economic evaluations of treatment and health care. The **Client Satisfaction Questionnaire-8** (CSQ-8) [37], which will be used to assess clients' satisfaction with the in-home decluttering program. The **Trimbos Questionnaire for Costs associated with Psychiatric Illness (TiC-P)** [38] will be used to assess healthcare and societal resource use.

Some of the measures will be administered as both clinician- and participant-rated outcomes. These secondary measures are the **Activities of Daily Living for Hoarding (ADL-H)** [39], measuring the degree to which hoarding symptoms impair daily functioning and the **Home Environment Index (HEI)** [22], measuring the sanitary state of the participant's homes. Since follow-up assessments will be carried out at the clinic, and not in the participant's homes, the ADL-H and the HEI will solely be administered as self-report forms during these assessment points.

The available relatives/caregivers of the participants will answer questions about demographic variables and will fill out the **Family Impact Scale for Hoarding Disorder (FISH)** [40], a scale that measures the level of family accommodation to the hoarding behaviours displayed by the relative who hoards and the burden these put on families, and the **Caregiver Burden Inventory (CBI)**, a non-hoarding specific measure [41].

In line with previous studies of HD [42, 43], responder status will be operationalized as clinically significant change on SI-R (change of 14 points or more) and remitter status will be operationalized as being a responder and have a total score of SI-R below a cut off of 42 [44].

Secondary outcome measures will generally be administered at pre-CBT, post-CBT, post in-home decluttering (week 10) and at 3-, 6- and 12-month follow-ups, with some exceptions (see Table 1 for full details). For all follow-up assessments, we will allow a -1 month/+2-month time period for the data collection.

Assessment point	*Telephone screening*	*Psychiatric assessment*	*Home assessment*	*Pre CBT*	*Mid CBT*	*Post CBT*<br><br>*(Baseline; pre in-home decluttering or wait list)*	*In-home decluttering Week 4 and 7*	*Post in-home*<br><br>*decluttering/ waitlist*<br><br>*(Primary endpoint)*	*Long term follow - ups*
**Clinician-rated outcomes**									
HRS-I	X								
MINI and OCD-RD		X							
SIHD		X	X [a]			X		X [b]	X
CIR			X [c]			X [c]	X [c]	X [c, d]	X [c, e]
ADL-H			X			X	X	X [f]	
HEI			X			X		X [f]	
CGI-S		X				X		X [d]	X
CGI-I						X		X [d]	X
SMURF								X [d]	
**Participant-rated outcomes**									
SI-R				X	X	X	X	X [f]	X
CIR			X		X	X	X	X	X
SCI		X		X	X	X		X [d]	X
CSQ-8						X		X [d]	X [g]
RSI			X			X		X [f]	X
EQ-5D		X				X		X [d]	X [g]
ADL-H				X		X	X	X [f]	X
HEI			X			X		X [f]	X
TiC-P		X				X		X [d]	

Table 1. Assessment points throughout the study.

Abbreviations: *HRS-I* Hoarding Rating Scale – Interview, *MINI* Mini International Neuropsychiatric Interview, *SIHD* Structured Interview for Hoarding Disorder, *CIR* Clutter Image Rating, *ADL-H* Activities of Daily Living for Hoarding, *HEI* Home Environment Index, *CGI-S* Clinical Global Impression Scale Severity, *CGI-I* Clinical Global Impression Scale Improvement, *SMURF* Safety Monitoring Uniform Report Form, *SI-R* Saving Inventory-Revised, *SCI* Saving Cognitions Inventory, *CSQ-8* Client Satisfaction Questionnaire-8, *RSI* Relationship between Self and Items, *EQ-5D* EuroQol-5D. *TiC-P* Trimbos Questionnaire for Costs associated with Psychiatric Illness.

The primary outcome measures are the blinded assessor-rated CIR and the participant-rated SI-R.
a. Only the risk section administered.
b. Administered/assessed at the clinic (during part 2 of the post in-home decluttering assessment).
c. Rated by blinded assessors.
d. Administered/assessed at the clinic (during part 2 of the post in-home decluttering assessment).
e. If participants provide pictures.
f. Administered/assessed at the last session (during part 1 of the post in-home decluttering assessment).
g. Administered only at the last follow-up timepoint (12 months).

7.1 END OF TRIAL

The trial will end when the final data (at the 12-month follow-up) has been collected for the final participant. The blinded part of the trial will end after the last participant has reached the primary endpoint.

7.2 PARTICIPANT WITHDRAWAL FROM TRIAL

Participants (and their relatives/carers) are free to withdraw from the trial at any point. After the withdrawal from treatment, participants will be asked to continue further trial measurements and to provide non-obligatory feedback regarding their reason(s) for withdrawal. The reason(s) (if given) will be logged for CONSORT reporting purposes. Previously collected data will be kept and this will be described in the consent form. Once participants have withdrawn from the trial, it will not be possible for them to re-enter. However, if there are strong clinical reasons, participants will be allowed to continue their CBT without participating in the trial.

Failure to complete outcome measures at one time point will not imply withdrawal from the trial, and the participants will still be invited to complete measures at the next time point, unless they explicitly state otherwise.

7.3 NONCOMPLIANCE AND DISCONTINUATION OF TRIAL

7.3.1 NONCOMPLIANCE

An instance of noncompliance during CBT-treatment will be defined as either missing a treatment session or completing less than 30% of that week's homework. The homework assignments will differ from session to session, but will for instance include reviewing the materials from the previous session, sorting and discarding items for half an hour a day and bringing objects from home to the session. Homework compliance will be rated by the psychologists during every treatment session using a worksheet in the treatment manual. Moreover, if a participant has 3 noncompliance sessions in a row, or 5 in total, the participant may be excluded from treatment, but kept in the trial. After the first instance of noncompliance, the participant will be reminded of the importance of attendance and homework completion. Subsequently, after a second instance of noncompliance, the participant will be offered a 5-10-minute session with one of the group leaders in order to problem-solve any barriers to complying with the requirements. After a third instance of noncompliance, the participant will be required to attend a separate meeting of 15-35 minutes with one of the group leaders to receive additional help with problem-solving barriers to complying and will be given the choice of continuing or withdrawing from the treatment. At the fourth instance of noncompliance, the participant will be required to meet with the PI or Co-PI before being allowed to attend any more group sessions. During in-home decluttering, we will apply a similar structure for handling noncompliance but the ratings will be conducted by the social workers and missing a session is defined as not letting the social workers in to your home The procedure for handling noncompliance above has previously been used in a trial testing the efficacy of the treatment protocol we will use in our study [12].

7.3.2 DISCONTINUATION OF TRIAL

Blind will not be broken and outcome measures will not be analyzed until the end of the controlled phase of the trial (when the last participant reaches the primary endpoint) and will therefore not inform decisions to stop the research. However, serious adverse events will be reviewed as the study is ongoing, and if there is any indication that these are linked to the intervention, consideration will be given to discontinuing the trial on the advice of the KTA and trial Sponsor.

7.4 QUALITY CONTROL

The trial will be monitored by KTA (Karolinska Trial Alians) using random samples of 15 % of the study participants. The KTA will monitor informed consent forms, identification log, journal entry of consent forms, inclusion/exclusion criteria, measures at primary endpoint (SI-R and CIR), and serious adverse events.

8 DATA MANAGEMENT

Data will be collected manually and online and kept in Case Report Forms (CRFs). The CRFs will not include the participant's name after the end of the trial, instead, the trial identification number will be used for identification. The CRFs will be stored securely in a locked archive at KI. The accuracy of the data entry for the primary outcome measure (SI-R) will be monitored by the KTA. The delegation log will identify all staff with responsibilities for data collection and handling, including those who have access to the trial database.

9 STATISTICAL ANALYSIS

9.1 SAMPLE SIZE CALCULATION AND STATISTICAL ANALYSIS

The power calculation in the current trial is based on individual-level data from a previous study of group CBT for HD with the SI-R as the primary outcome measures rated at 3 time points [18]. To calculate the required sample size, we used a linear, 2-level mixed effects model (LMM) implemented using the R-package **powerlmm** version 0.4 (available at https://cran.r-project.org/web/packages/powerlmm/index.html)[45]. In this model, we imputed the standard deviations of the random intercept (4.22), the random slope (2.54), and the residual error (5.68). Given 80% power (two-sided alpha test), two groups (in-home decluttering and waitlist) and 4 observations (SI-R) per participant, we estimated that a total of 80 participants will be needed to detect a slope difference between the two groups of 8 points on the SI-R at the primary endpoint. For the second primary outcome measures, the CIR, we used data from the same study as above. As we only had data from two data points, we used an ANCOVA in which we adjusted for pre-treatment scores. Given 80% power (two-sided alpha test), two groups (treatment and waitlist) and 4 observations (CIR) per participant, we estimated that a total of 58 participants will be needed to detect a slope difference between the two groups of 1 point on the CIR at the primary endpoint.

In order to compensate for 15% missing data throughout the trial, we thus aim to recruit a total of 90 participants that will be randomized to one of two trial arms. Because we expect to lose

some participants after the first phase of the study (group CBT), we plan to recruit patients to CBT until we have randomized 90 participants.

All data will be analyzed according to intention-to-treat principles with mixed effects models. The mixed effect regression model will include fixed effects of time, trial arm (treatment vs waitlist) and an interaction effect of trial arm by time, as well as random intercept to account for individual differences. Ordinal variables (such as CGI-I and CGI-S) will be analyzed with ordinal regression and binary data (e.g. remitter status) with logistic regression. Chi-square tests will be used to check whether the blinded assessor's guesses on trial arm allocation are better than chance. The statistician that will perform the data analyses will be independent to the research group and blind to group allocation.

9.2 COST-EFFECTIVENESS ANALYSIS

In our cost-effectiveness analysis, we will calculate the costs and health outcomes of in-home decluttering, compared to the waitlist. Costs will be collected from a social services provider perspective. Such costs include recorded time for home visits (including time for preparation and traveling), telephone calls, and administration. We will conduct two types of analyses; a cost-effectiveness analysis and a cost-utility analysis. In the cost-effectiveness analysis, hoarding symptoms, responder and remitter status, and level of clutter, measured with SI-R and CIR respectively, at the primary endpoint (post in-home decluttering) will be used as the health outcome. In the cost-utility analysis, the outcome will be the quality-adjusted life years (QALYs), according to international standards for cost-effectiveness analyses [46]. QALYs will be calculated using the area under the curve method. Cost-effectiveness planes will be presented from 3 perspectives: (A) social services provider perspective, (B) social services sector perspective, and (C) societal perspective. Costs will be estimated by appropriate regression analyses testing alternative link functions and distributions. Non-parametric bootstrapping with 1000 iterations will be carried out, pairing up differences in costs with differences in outcomes. As a global cost-effectiveness estimate, we will present a visual presentation of cost-effectiveness planes. The timeframe for the cost-effectiveness analyses will be from baseline to post in-home decluttering.

9.3 THE PROCESS EVALUATION

In our process evaluation, we will follow the MRC[47] directives for process evaluations of complex interventions using a mixed-methods design, combining qualitative and quantitative data from the trial. All interviews will be recorded with a dictaphone and then transcribed verbatim. In the subsequent step, thematic analysis will be used to identify, analyze, and report patterns within the interviews. A form of thematic analysis named the Framework Method (KÄLLA?) of analysis will be employed, as it is the most commonly used method for thematic analysis of semi-structured interviews. The quantitative data such as total social worker time, number of home-sessions, and symptom outcome results will first be analyzed separately from the qualitative data and in the second step, mixed with the methodological approach triangulation [48]. Equal importance will be given to both types of data as they are equally important for addressing the research questions of the process evaluation.

10 RECORDING AND REPORTING OF ADVERSE EVENTS

Data on adverse events will be collected using a standardized checklist (Safety Monitoring Uniform Report Form, SMURF), see Table 1 for assessment points. If the researcher team would be notified about adverse events at other times, this will be logged and, if needed, an assessment about continued participation in the study will be made. If a participant expresses suicidal ideation or worsening of symptoms is suspected, a clinical assessment with a structured suicide risk evaluation will be offered as soon as possible, as well as an in-depth assessment of the symptoms. If there is an urgent need for psychiatric care, the participant will get an appointment with a psychiatrist that works in the clinic.

Reported adverse events will be categorized depending on severity and frequency in line with GCP principles. All adverse event assessments will be crosschecked by KTA. All participants will be monitored by the study personnel throughout the study period to ensure participant safety. Serious adverse events (i.e., events that result in death, suicide attempt, serious violent incident or admission to hospital) will be documented and monitored by KTA.

11 PUBLIC AND PATIENT INVOLVEMENT

This trial builds upon previous feedback from therapists, social workers, patients, and patient organizations with a desire to improve collaboration between healthcare and municipalities.

The Task Force for Hoarding Disorder in Stockholm has been and will continue to be a collaborating partner in this project. This ensures that experience from every phase of the project is directly communicated to all relevant interested parties (including patient representatives). As the task force includes a broad range of members, it also represents a crucial resource for identifying individuals with HD in the broader community who would benefit from treatment but not otherwise seek it out. Moreover, patients will be represented by the Swedish OCD patient organization (https://ocdforbundet.se/) who are members in the task force, making sure that the patient perspective is acknowledged throughout all phases of the project.

No patients have been involved in the current design of the study or in the decision of outcome measures. Patients that have received treatment before the study have continuously had the opportunity to provide feedback about their experiences. As a result of this feedback, some small modifications to the CBT workbook in Swedish have been made (e.g., simplifying and making worksheets easier to use and using the word ‘treatment’ instead of ‘course’). The patient organization for OCD and related disorders in Stockholm (OCD-föreningen Stockholms län) provided feedback on the current design and some modifications to exclusion criteria were made after their notes. For instance, the exclusion criterion having received previous treatment for HD was changed and currently, only previous treatments within 2 years render exclusion from the trial. Moreover, caregiver burden among relatives to participants in the trial was given greater consideration and included as one of the treatment targets in the trial.

We plan to disseminate the results of the research to study participants, the Swedish OCD Foundation (*Svenska OCD-förbundet*), members of the task force, politicians and the general public.

12 DATA SHARING

We will potentially share trial data with other HD researchers around the world in accordance with laws and local regulations and whenever data sharing is permitted. The use will primarily be for similar objectives as in the current trial, for instance to combine treatment outcome data for meta-analyses. Shared data will be pseudonymized. The possibility of future data sharing is mentioned in the informed consent form.

13 ETHICAL CONSIDERATIONS

Our clinical point of view is that the proposed combined interventions pose a small risk to participants. All patients screened for inclusion in the study will receive a thorough clinical assessment and those with more immediate needs or risks will be offered alternative treatment options. No patients will be denied CBT (current standard treatment) due to drop-out from the study or if they do not want to participate at all. Participants may withdraw from the trial at any time. The CBT treatment offered in this trial only differs in that it includes fewer sessions than the original manual, but there is evidence that treatments including even fewer sessions are acceptable [25]. Moreover, as the participants are further offered 10 additional in-home sessions, we cannot identify any risks associated with altering the treatment length.

Adverse events will be carefully monitored throughout the trial but the risk of serious adverse events due to the interventions is considered very low. The adverse events that may be experienced in the short term, such as anxiety due to discarding belongings, are difficult to avoid and are often necessary for the participants to reduce their hoarding difficulties. Moreover, the in-home decluttering intervention may be perceived as a privacy violation, since the work will take place in the participants' home and pictures of their home will be taken for the blinded measures of clutter. This is not a risk that is solely linked to this study, it also occurs in conventional outreach housing support and is considered difficult to circumvent. The in-home decluttering team is used to working under these circumstances and to preventing the potential experience of a privacy violation as much as possible.

Participants randomized to waitlist after CBT will receive home visits after 10 weeks (in some cases later due to vacations during summer). Although this delay in support can be perceived as aversive by some participants, the duration of the delay should be considered as clinically ethical in light of the otherwise chronic course of HD.

We will also take several measures to improve the scientific quality of the study and improve dissemination of the new knowledge, for example, registering the statistical analysis plan (ClinicalTrials.gov) prior to the inclusion of the first participant, making all statistical code publicly available, and publishing the final report as an open access article. The study will also follow local regulation for data management and protection, the Swedish law, and the General Data Protection Regulation (GDPR).

13.1 THE COVID-19 PANDEMIC

This trial will follow the recommendations of the Swedish public health authority (Swedish: *Folkhälsomyndigheten*) and adapt/modify procedures according to these, if relevant/necessary. Any changes to the protocol will be documented and amendments submitted for approval to the ethics committee.

14 IMPLICATIONS

The complex consequences of the disorder mean that individuals with HD often require care from both mental health and social services. However, a coordinated intervention from both these services is currently unavailable, leading to prolonged suffering and increased societal costs. Joining Forces has the potential to offer a coordinated intervention between healthcare and social services for HD.

The immediate clinical impact, if our hypotheses are confirmed, is that the study results can guide implementation of specialized support for HD in the Stockholm municipality. Since clinical services and stakeholders are already involved in the project, implementation is expected to be straightforward. If our hypotheses are confirmed, this will also highlight the need to further develop this coordinated intervention to help more people suffering from HD. This could help decrease societal costs, suffering among relatives, and increase the feeling of being able to provide efficacious help among personnel in both social services and the healthcare system.

Furthermore, the PI has a broad network of municipalities all over Sweden through which the study results can be disseminated nationally. Moreover, as the project is an international collaboration, we will share our results with several research groups in the U.S., increasing their visibility and impact internationally.

15 FINANCE

This trial is financed by the Swedish Research Council for Health, Working Life and Welfare, FORTE (grant number 2019-01637).

16 PUBLICATIONS

We will communicate our results to the scientific community by publishing the results in high impact scientific journals. In order to maximize visibility of our results, we will pursue open access formats. Additionally, results will be presented at a number of national and international key conferences delivering results directly to active members of the scientific community. (e.g., American Psychiatric Association), and other professional venues.

17 CLINICAL INFRASTRUCTURE

The research group responsible for the trial is led by Christian Rück, based within the Stockholm Health Care Services and closely linked to the Center for Psychiatry Research (CPF) and located in the Stockholm with adult outpatient services (*OCD-programmet),* providing a fertile environment for clinical research. Clinicians work alongside researchers

with the common goal to develop, test, and make available evidence-based treatments for people with mental illnesses in the Stockholm county, as well as contributing to national and international development of the field. Members of our research network have pioneered developing internet-delivered CBT treatment protocols (e.g., panic disorder, social phobia, obsessive-compulsive disorder and body dysmorphic disorder), conducting RCTs and implementing them in the health care system within Stockholm since 2007, and making the treatments available nationally. The infrastructure for all of the most crucial parts of this study such as recruitment, data collection, and quality control (including techniques to limit data loss) are therefore existing within the research group. During the trial, the research group will also build on the collaboration with the social services unit delivering he in-home decluttering with which we have been collaborating and offering supervision for HD cases during the past 3 years.

18 REFERENCES

1. American Psychiatric Association, *Diagnostic and Statistical Manual of Mental Disorders*. 5th ed. 2013, Washington, DC.
2. Khoury, B., C. Kogan, and S. Daouk, *International Classification of Diseases 11th Edition (ICD-11)*, in *Encyclopedia of Personality and Individual Differences*, V. Zeigler-Hill and T.K. Shackelford, Editors. 2017, Springer International Publishing: Cham. p. 1-6.
3. Postlethwaite, A., S. Kellett, and D. Mataix-Cols, *Prevalence of Hoarding Disorder: A systematic review and meta-analysis.* J Affect Disord, 2019. **256**: p. 309-316.
4. Bratiotis, C., *Community hoarding task forces: a comparative case study of five task forces in the United States.* Health Soc Care Community, 2013. **21**(3): p. 245-53.
5. Ong, C., et al., *Functioning and quality of life in hoarding: A systematic review.* Journal of Anxiety Disorders, 2015. **32**: p. 17-30.
6. Luu, M., et al., *Squalor in community-referred hoarded homes.* Journal of Obsessive-Compulsive and Related Disorders, 2018. **19**: p. 66-71.
7. Rodriguez, C.I., et al., *Prevalence of hoarding disorder in individuals at potential risk of eviction in New York City: a pilot study.* J Nerv Ment Dis, 2012. **200**(1): p. 91-4.
8. Frost, R.O., G. Steketee, and L. Williams, *Hoarding: a community health problem.* Health Soc Care Community, 2000. **8**(4): p. 229-234.
9. Drury, H., et al., *Caregiver burden, family accommodation, health, and well-being in relatives of individuals with hoarding disorder.* J Affect Disord, 2014. **159**: p. 7-14.
10. Steketee, G., et al., *Waitlist-controlled trial of cognitive behavior therapy for hoarding disorder.* Depress Anxiety, 2010. **27**(5): p. 476-84.
11. Mathews, C.A., et al., *Randomised clinical trial of community-based peer-led and psychologist-led group treatment for hoarding disorder.* BJPsych Open, 2018. **4**(4): p. 285-293.
12. Tolin, D.F., et al., *Efficacy and mediators of a group cognitive-behavioral therapy for hoarding disorder: A randomized trial.* J Consult Clin Psychol, 2019. **87**(7): p. 590-602.
13. Tolin, et al., *Cognitive behavioral therapy for hoarding disorder: a meta-analysis.* Depress Anxiety, 2015. **32**(3): p. 158-66.
14. Reports, S.G.O., *Vad Är Psykiskt Funktionshinder?[What Is a Psychiatric Disability?] Rapport 2006: 5 Från Nationell Psykiatrisamordning*. 2006, National Board of Health and Welfare Stockholm, Sweden.
15. Brunt, D. and L. Tibblin, *Supported housing and housing support for the psychiatrically disabled – Background, population, policies, practices and current challenges.* 2016, 2016. **23**(1-2): p. 12.
16. Bodryzlova, Y. and K. O’Connor, *Factors Affecting the Referral Rate of the Hoarding Disorder at Primary Mental Health Care in Quebec.* Community Mental Health Journal, 2018. **54**(6): p. 773-781.
17. Tolin, D.F., Worden, B. L., Wootton, B. M., & Gilliam, C. M., *A cognitive-behavioral group treatment manual for hoarding disorder: Therapist manual.* 2017, West Sussex, UK:: Wiley-Blackwell.
18. Ivanov, V.Z., et al., *Enhancing group cognitive-behavioral therapy for hoarding disorder with between-session Internet-based clinician support: A feasibility study.* J Clin Psychol, 2018.
19. Rodriguez, C.I., et al., *Acceptability of Treatments and Services for Individuals with Hoarding Behaviors.* J Obsessive Compuls Relat Disord, 2016. **11**: p. 1-8.
20. Linkovski, O., et al., *Augmenting Buried in Treasures with in-home uncluttering practice: Pilot study in hoarding disorder.* J Psychiatr Res, 2018. **107**: p. 145-150.
21. Tolin, D.F., R.O. Frost, and G. Steketee, *A brief interview for assessing compulsive hoarding: the Hoarding Rating Scale-Interview.* Psychiatry Res, 2010. **178**(1): p. 147-52.
22. Rasmussen, J.L., et al., *Assessing Squalor in Hoarding: The Home Environment Index.* Community Mental Health Journal, 2014. **50**(5): p. 591-596.
23. Nordsletten, A.E., et al., *The Structured Interview for Hoarding Disorder (SIHD): Development, usage and further validation.* Journal of Obsessive-Compulsive and Related Disorders, 2013. **2**(3): p. 346-350.

24. Frost, R.O., et al., *Development and validation of the clutter image rating.* Journal of Psychopathology and Behavioral Assessment, 2008. **30**(3): p. 193-203.
25. Moulding, R., et al., *Short-Term Cognitive-Behavioural Group Treatment for Hoarding Disorder: A Naturalistic Treatment Outcome Study.* Clin Psychol Psychother, 2017. **24**(1): p. 235-244.
26. Sheehan, D.V., et al., *The Mini-International Neuropsychiatric Interview (M.I.N.I.): the development and validation of a structured diagnostic psychiatric interview for DSM-IV and ICD-10.* J Clin Psychiatry, 1998. **59 Suppl 20**: p. 22-33;quiz 34-57.
27. Iervolino, A.C., et al., *Prevalence and heritability of compulsive hoarding: a twin study.* Am J Psychiatry, 2009. **166**(10): p. 1156-61.
28. Svanborg, P. and M. Asberg, *A new self-rating scale for depression and anxiety states based on the Comprehensive Psychopathological Rating Scale.* Acta Psychiatr Scand, 1994. **89**(1): p. 21-8.
29. Saunders, J.B., et al., *Development of the Alcohol Use Disorders Identification Test (AUDIT): WHO Collaborative Project on Early Detection of Persons with Harmful Alcohol Consumption--II.* Addiction, 1993. **88**(6): p. 791-804.
30. Berman, A.H., et al., *Evaluation of the Drug Use Disorders Identification Test (DUDIT) in criminal justice and detoxification settings and in a Swedish population sample.* Eur Addict Res, 2005. **11**(1): p. 22-31.
31. Frost, R.O., G. Steketee, and J. Grisham, *Measurement of compulsive hoarding: saving inventory-revised.* Behav Res Ther, 2004. **42**(10): p. 1163-82.
32. Guy, W., *Clinical global impression scale.* 1976.
33. Steketee, G., R.O. Frost, and M. Kyrios, *Cognitive Aspects of Compulsive Hoarding.* Cognitive Therapy and Research, 2003. **27**(4): p. 463-479.
34. Dozier, M.E., et al., *A Preliminary Investigation of the Measurement of Object Interconnectedness in Hoarding Disorder.* Cognitive Therapy and Research, 2017. **41**(5): p. 799-805.
35. Aron, A., E.N. Aron, and D. Smollan, *Inclusion of Other in the Self Scale and the structure of interpersonal closeness.* Journal of Personality and Social Psychology, 1992. **63**(4): p. 596-612.
36. Rabin, R. and F.d. Charro, *EQ-SD: a measure of health status from the EuroQol Group.* Ann. Med., 2001. **33**(5): p. 337-343.
37. Attkisson, C.C. and R. Zwick, *The client satisfaction questionnaire. Psychometric properties and correlations with service utilization and psychotherapy outcome.* Eval Program Plann, 1982. **5**(3): p. 233-7.
38. Bouwmans, C., et al., *Feasibility, reliability and validity of a questionnaire on healthcare consumption and productivity loss in patients with a psychiatric disorder (TiC-P).* BMC health services research, 2013. **13**: p. 217-217.
39. Frost, R.O., et al., *Activities of Daily Living Scale in Hoarding Disorder.* J Obsessive Compuls Relat Disord, 2013. **2**(2): p. 85-90.
40. Nordsletten, A., et al., *The Family Impact Scale for Hoarding (FISH)* ©. 2014.
41. Novak, M. and C. Guest, *Application of a Multidimensional Caregiver Burden Inventory1.* The Gerontologist, 1989. **29**(6): p. 798-803.
42. Mathews, C.A., et al., *Randomised clinical trial of community-based peer-led and psychologist-led group treatment for hoarding disorder.* BJPsych Open, 2018. **4**(4): p. 285-293.
43. Frost, R.O., D. Ruby, and L.J. Shuer, *The buried in treasures workshop: Waitlist control trial of facilitated support groups for hoarding.* Behaviour Research and Therapy, 2012. **50**(11): p. 661-667.
44. Frost, R.O. and V. Hristova, *Assessment of hoarding.* J Clin Psychol, 2011. **67**(5): p. 456-66.
45. Galbraith, S., M.Stat, and I.C. Marschner, *Guidelines for the design of clinical trials with longitudinal outcomes.* Controlled Clinical Trials, 2002. **23**(3): p. 257-273.
46. Bridges, J.F., et al., *Conjoint analysis applications in health--a checklist: a report of the ISPOR Good Research Practices for Conjoint Analysis Task Force.* Value Health, 2011. **14**(4): p. 403-13.
47. Moore, G.F., et al., *Process evaluation of complex interventions: Medical Research Council guidance.* BMJ : British Medical Journal, 2015. **350**: p. h1258.

48. O'Cathain, A., E. Murphy, and J. Nicholl, *Three techniques for integrating data in mixed methods studies*. BMJ, 2010. **341**: p. c4587.
